# A quantitative multimodal metabolomic assay for colorectal cancer

**DOI:** 10.1186/s12885-017-3923-z

**Published:** 2018-01-04

**Authors:** Farshad Farshidfar, Karen A. Kopciuk, Robert Hilsden, S. Elizabeth McGregor, Vera C. Mazurak, W. Donald Buie, Anthony MacLean, Hans J. Vogel, Oliver F. Bathe

**Affiliations:** 1 Department of Surgery, University of Calgary, Calgary, AB Canada; 2 Department of Oncology, University of Calgary, Calgary, AB Canada; 3 Department Mathematics and Statistics, University of Calgary, Calgary, AB Canada; 4 Department of Medicine, University of Calgary, Calgary, AB Canada; 5 Department of Biological Sciences, University of Calgary, Calgary, AB Canada; 6 Forzani & MacPhail Colon Cancer Screening Centre, Calgary, AB Canada; 7 Population Health Research, Alberta Health Services, Calgary, AB Canada; 8 Department of Agricultural, Food and Nutritional Science, University of Alberta, Edmonton, Canada; 9 Division of Surgical Oncology, Tom Baker Cancer Centre, 1331 – 29th St NW, Calgary, AB T2N 4N2 Canada

**Keywords:** Colorectal cancer, Metabolomics, Colorectal adenoma, Colorectal adenocarcinoma, Metabolomics profiling, Mass spectrometry, Cancer biomarker

## Abstract

**Background:**

Early diagnosis of colorectal cancer (CRC) simplifies treatment and improves treatment outcomes. We previously described a diagnostic metabolomic biomarker derived from semi-quantitative gas chromatography-mass spectrometry. Our objective was to determine whether a quantitative assay of additional metabolomic features, including parts of the lipidome could enhance diagnostic power; and whether there was an advantage to deriving a combined diagnostic signature with a broader metabolomic representation.

**Methods:**

The well-characterized Biocrates P150 kit was used to quantify 163 metabolites in patients with CRC (*N* = 62), adenoma (*N* = 31), and age- and gender-matched disease-free controls (*N* = 81). Metabolites included in the analysis included phosphatidylcholines, sphingomyelins, acylcarnitines, and amino acids. Using a training set of 32 CRC and 21 disease-free controls, a multivariate metabolomic orthogonal partial least squares (OPLS) classifier was developed. An independent set of 28 CRC and 20 matched healthy controls was used for validation. Features characterizing 31 colorectal adenomas from their healthy matched controls were also explored, and a multivariate OPLS classifier for colorectal adenoma could be proposed.

**Results:**

The metabolomic profile that distinguished CRC from controls consisted of 48 metabolites (R^2^Y = 0.83, Q^2^Y = 0.75, CV-ANOVA *p*-value < 0.00001). In this quantitative assay, the coefficient of variance for each metabolite was <10%, and this dramatically enhanced the separation of these groups. Independent validation resulted in AUROC of 0.98 (95% CI, 0.93–1.00) and sensitivity and specificity of 93% and 95%. Similarly, we were able to distinguish adenoma from controls (R^2^Y = 0.30, Q^2^Y = 0.20, CV-ANOVA *p*-value = 0.01; internal AUROC = 0.82 (95% CI, 0.72–0.93)). When combined with the previously generated GC-MS signatures for CRC and adenoma, the candidate biomarker performance improved slightly.

**Conclusion:**

The diagnostic power for metabolomic tests for colorectal neoplasia can be improved by utilizing a multimodal approach and combining metabolites from diverse chemical classes. In addition, quantification of metabolites enhances separation of disease-specific metabolomic profiles. Our future efforts will be focused on developing a quantitative assay for the metabolites comprising the optimal diagnostic biomarker.

**Electronic supplementary material:**

The online version of this article (10.1186/s12885-017-3923-z) contains supplementary material, which is available to authorized users.

## Background

Colorectal cancer (CRC) is the third most common cause of cancer death worldwide. Early diagnosis is important, as early disease is curable [1–3] and management of the more advanced disease is more complex as well as more morbid. Currently screening efforts hinge on colonoscopy and fecal occult blood tests. Colonoscopy is extremely sensitive, and it is capable of detecting (and treating) very early premalignant disease. However, it is invasive and difficult to apply to large populations in an efficacious manner. Fecal tests have been used to enrich the population that should undergo colonoscopy. Unfortunately, their implementation as a broadly applied screening tests is hampered by low patient acceptance and low compliance. A single blood test for CRC may be more acceptable to patients, and could potentially represent an attractive alternative, especially if it is more sensitive than fecal tests.

We have previously identified a number of changes in sugar derivative structures, amino acids, and short-chain fatty acids in the serum metabolome in association with CRC [4, 5]. These validated changes were identified using gas chromatography-time of flight-mass spectrometry (GC-TOF-MS), and they could represent the basis for a future blood test. In this work, we sought to determine whether a more comprehensive coverage of the metabolome may enhance the performance of this diagnostic test. Specifically lipid-consisting molecules, including lipoproteins have been shown by others to be altered in the serum of patients with CRC [6, 7], and so we postulated that these perturbations would further inform a diagnostic metabolomic biomarker for CRC.

Our objective was to determine whether a fully quantitative metabolomic test enhances one’s capability to discriminate between disease states. To this end, we profiled CRC using the quantitative mass spectrometry-based metabolomic Biocrates assay. The assay provided measurements of a broad array of endogenous lipids (including glycerophospholipids (phosphatidylcholines and lysophosphatidylcholines), sphingolipids) and acylcarnitines, as well as 14 amino acids. In addition to exploring the potential value of this quantitative approach to the development of a diagnostic test, the biological implications of our findings were evaluated. Finally, we assessed whether the addition of these metabolites to the diagnostic model that was previously devised using GC-MS profiling [5] would add to the diagnostic power of the metabolomic profile.

## Methods

### Sample collection

This study was approved by the Conjoint Health Research Ethics Board at the University of Calgary (Ethics ID number E21805) and conforms to the Helsinki Declaration (October 2008). Colorectal cancer serum samples from patients in stage I, stage II, and stage III (locoregional CRC), and stage IVa (liver-limited metastasis) along with the relevant clinical information were collected in a prospective cohort of colorectal cancer patients, diagnosed at the Foothills Medical Center or referred to this center for resection and management of their primary adenocarcinoma or metastatic CRC and provided written consent, between 2006 and 2012. Patients with extrahepatic metastases, any acute inflammatory state, sepsis, and familial colorectal adenomatosis or cancer cases were excluded from this study. Colorectal adenoma samples and control samples were collected prospectively by the Forzani & MacPhail Colon Cancer Screening Centre (CCSC) at the University of Calgary. Disease-free controls consisted of individuals who underwent screening colonoscopy and were found not to have CRC or adenoma. CRC, adenoma, and control cases were all fasting for a minimum of 8 h before surgery or endoscopic procedure. Surgical pathology for CRC patients and endoscopic pathology for adenoma and control cases were obtained and thoroughly reviewed, and all diagnoses were confirmed. Control samples are matched for age and gender with adenoma and cancer patients. Samples were collected in plastic gold top vacutainer tubes (BD Biosciences, Mississauga, Ontario, Canada), which contain a clot activator and a proprietary gel for serum separation. Samples were processed in 6-h time from collection and were stored in −20°c freezers until the day of analysis [8, 9].

### Quantitative profiling by flow injection analysis-tandem mass spectrometry (FIA-MS/MS)

In these studies, we used quantitative Biocrates Absolute*IDQ* p150 Kit (Biocrates Life Sciences AG, Austria) which can measure the concentration of 163 endogenous metabolites from 4 biochemical classes, using a panel of 27 validated internal controls. These metabolites are selected from four classes of acylcarnitines, biogenic amines, sphingolipids, and glycerophospholipids (lyso-phosphatidylcholines and phosphatidylcholines). Detailed preparation steps have been previously described [10]. In brief, 10 μl of each serum sample was prepared and submitted to mass spectrometric analysis on an API4000 Qtrap® tandem mass spectrometry instrument (Applied Biosystems/MDS Analytical Technologies, Foster City, CA) equipped with a solvent delivery system, in both positive and negative ion modes. Selective multiple reaction monitoring (MRM) detections of chemical homologs in conjunction with stable isotope-labeled internal controls, provided in the kit plate filter, was utilized for metabolite quantification. This method is shown to be in compliance with the FDA guidance for industry- Bioanalytical method validation [11], indicating proof of reproducibility in the given range. External quality control samples consisted of four commercially available human serum samples (Sigma-Aldrich, Germany) as well as four pooled serum samples from our controls for each kit. The final metabolite concentrations were automatically calculated by Met*IDQ*, the proprietary software of the kit. Metabolites were checked for the coefficient of variance >0.25 in quality controls, and all passed the check. The concentrations of 146 metabolites were above the limit of detection, and these metabolites were selected for further analysis. Missing values were imputed with the minimum value in the dataset. The pre-processed data were then transferred for further statistical analyses.

### Data analysis

Throughout this study, wherever a two group statistical comparison were desired, a two-sided Student’s t-test was used. We considered a priori *p*-value smaller than 0.05 as statistically significant. Where required, the significance thresholds adjusted by Holm-Bonferroni correction method were used. For analysis of stage-dependent variations in more than two groups, Bonferroni-corrected Kruskal-Wallis Test [8] (non-parametric approach) was computed by Multi-Experiment Viewer (MeV), version 4.9 (The TM4 Software Development Team) [9]. To generate heatmaps, we used the Spearman’s Rank Correlation distance metrics and complete linkage method.

The pre-processed data from MetIDQ were log-transformed and autoscaled (unit variate scaled and centered) before importing the SIMCA multivariate analytical software (Version 14.0.0, Umetrics AB, Sweden). Owing to the quantitative nature of the assay, we require median fold change normalization to account for inter-sample analytical biases. To evaluate the risk of overfitting bias on a dataset with small sample size, a pre-analysis permutation test of 10,000 iterations was applied on sample classes in the training set, as described before by Westerhuis et al. [12]. As for the combinatorial analysis, the relevant datasets were joined and then block-transformed [13–16]. For each comparison, an exploratory Principal Component Analysis (PCA) with up to three components was used for discovering intrinsic clusters and revealing potential outliers. After exclusion of outliers, subsets of potentially significant metabolites for each comparison were selected by performing Welch’s *t*-test (assuming unequal variances). This filtering procedure was performed by setting a pre-test maximum *p*-value threshold of 0.30 in the Welch test, which removes clearly uniformative metabolites from further analysis [4, 17, 18]. Selected subsets were used for orthogonal partial least squares discriminant analysis (OPLS-DA) or O2-PLS-DA. Further refinement was applied through excluding metabolites with variable importance on projection (VIP) of less than a threshold. This VIP threshold was set separately for each analysis, so as the maximum for R^2^Y and Q^2^Y are obtained and their difference is at minimum. This approach has shown to be sufficiently reliable for the purpose of multivariate statistical analyses, including OPLS-DA [4, 17].

To assess the performance of supervised multivariate models, including OPLS-DA and O2PLS-DA, R^2^Y and Q^2^Y scores were used for measurement of the dataset variance covered by the model, and the predictability of the model in 7-fold cross-validation [4]. Models with the difference of more than 0.2 between R^2^Y and Q^2^Y were reevaluated. Potential confounders in each model were evaluated for their unwanted effects, as described in the Results section. Also, to examine the PLS-DA and OPLS-DA models for validity and potential overfit, a permutation test of 999 iterations was applied to each model, and the results were reported as Q2-intercept for that model [19]. Q2-intercept is the intercept of a line fitted to Q^2^Y scores versus the correlation of the permutated Y-vector and original Y-vector for each iteration. The model is valid and non-random if Q2 intercept is at or below zero [20].

Predictive performance of the generated models in external validation were evaluated by the area under the receiver operating characteristic curves (AUROC), which were calculated by GraphPad Prism (version 6.01 for Windows, GraphPad Software, La Jolla California USA, www.graphpad.com).

## Results

### Patients and demographics

The characteristics of the study cohort are summarized in Table 1. Samples were randomly assigned to the training set and validation set, in a stratified design for locoregional CRC (stages I, II, and III), and liver-limited metastatic CRC (stage IVa). In patients with stage IVA disease, 17 (45%) received chemotherapy within 3 months before sampling; 8 patients (33%) with non-metastatic disease received chemotherapy before sampling.

**Table 1:** Demographics and clinical factors of patients and controls. Numbers in parentheses represent percent, unless otherwise noted. Disease-free controls (*N* = 31) were matched with the adenoma cases, and 50 controls were matched with patients with CRC

	Subgroup	Control	Adenoma	Stage I	Stage II	Stage III	Stage IVa
N		31 + 9 + 41	31	8	8	8	38
Age, mean (SD)		60.5 (6.7)	59.5 (5.9)	76.9 (6.1)	67.3 (9.8)	62.8 (14.8)	60.5 (9.9)
Gender	Male	58 (72)	21(68)	5 (63)	7(88)	4 (50)	30 (79)
	Female	23 (28)	10 (32)	3 (37)	1 (11.2)	4 (50)	8 (11)
Primary Site	Colon	–	–	2	3	5	–
	Rectum	–	–	5	4	3	–
	Unknown	–	–	1	1	0	–
Differentiation	Poor	–	–	0	1	2	3
	Moderate	–	–	4	5	4	26
	Well	–	–	2	0	2	0
	Unspecified	–	–	2	2	0	7 (+2 mucinous)
Staging T	T1	–	–	1	–	3	–
	T2	–	–	5	–	2	–
	T3	–	–	–	7	1	–
	T4	–	–	–	0	0	–
	Unspecified	–	–	2	1	2	
Staging N	N1	–	–	–	–	4 (N1a = 2) (N1b = 2)	8
	N2	–	–	–	–	2 (N2a = 1) (N2b = 1)	8
	Unspecified	–	–	–	–	2	22
Tumor dimension (cm)	Largest dimension, mean (SD)	–	–	2.67 (1.50)	3.51 (1.99)	2.7 (2.09)	2.50 (1.49)
	Min-Max			0.5–5	2.5–6	2.8–5.5	0.9–5.1
Pre-sampling Chemotherapy		–	–	1 (13)	3 (38)	4 (50)	17 (45)

### Identification of metabolites associated with CRC

**Fig. 1:**
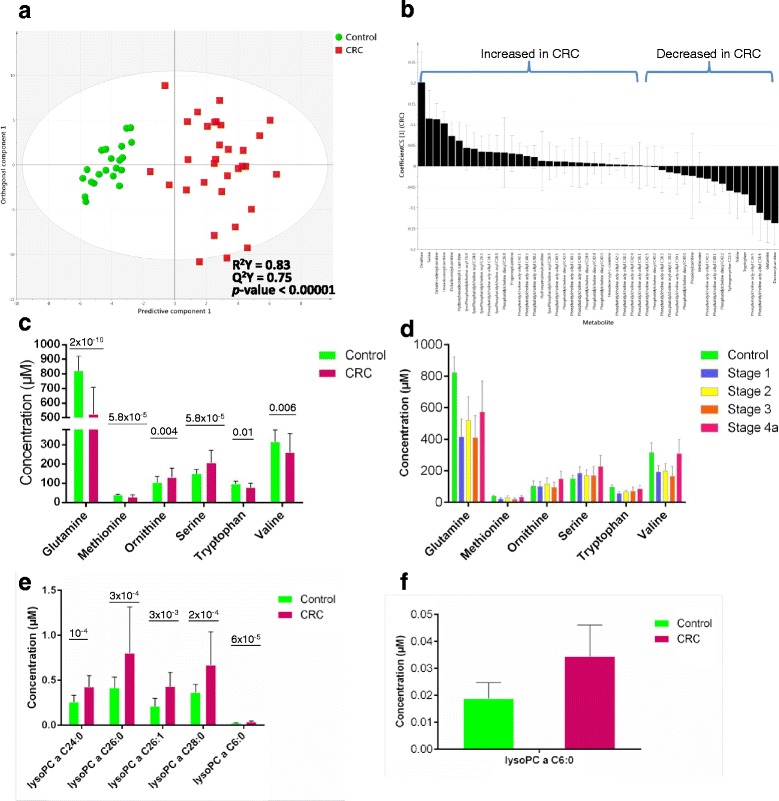
Metabolomic profile as determined by targeted MS/MS. **a** Supervised (OPLS-DA) analysis scores scatter plot illustrating that the metabolomic profile of CRC is distinct from matched controls. **b** Coefficient column plot for OPLS-DA analysis of CRC vs. matched controls. **c** Amino acids differentially abundant in CRC vs. control cases, **d** Amino acids concentrations as a function of stage of disease. **e** Lysophosphatidylcholines differentially abundant in CRC serum samples. **f** Lysophosphatidylcholine C6:0 (magnified from Figure 1e). Details of concentrations, significance testing *p*-values, and other characteristics are described in Additional file 1: Tables S1 and S2

To quantitatively evaluate serum composition of amino acids, acylcarnitines, and lipid compounds, including glycerophospholipids and sphingolipids, we submitted samples to semi-quantitative FIA-MS/MS. Out of 163 measured metabolites, 146 metabolites could be reliably found in all groups of patients and controls (Additional file 1: Table S1). Three samples were identified as outliers on PCA, and they were excluded from further analysis. We also evaluated for a potential confounding effect of pre-sampling chemotherapy on the metabolomic profile of CRC patients (Additional file 2: Fig. S1A to E). We could not identify any cluster linked to the chemotherapy status. The training set consisted of 53 samples (controls (*N* = 21), stage I (*N* = 5), stage II (*N* = 5), stage III (*N* = 5) and stage IVa (*N* = 17) cases). Following filtering by *p*-value and VIP (>1), 48 metabolites were employed in the metabolomic model, which resulted in an encouraging model: R^2^Y was 0.83 and Q^2^Y was 0.75; CV-ANOVA was <0.00001 (Fig. 1a and b, and Table 2). The model was then tested on an independent validation set (controls (*N* = 20), stage I (*N* = 3), stage II (*N* = 3), stage III (*N* = 3) and stage IVa (*N* = 19) cases). Sensitivity in this external validation set was 93%, and specificity was 95%; accuracy was 94%, and precision was 96%; AUROC was 98%.

**Table 2:** List of 48 metabolites in targeted MS/MS analysis of colorectal cancer vs. healthy controls. Variable importance in projection (VIP) and multivariate correlation coefficients are provided (PC: Phosphatidylcholine, Lyso-PC: lysophosphatidylcholine, ae: acyl-alkyl, aa: diacyl, SM: sphingomyelin)

Abbreviated name	Metabolite	VIP	Centered and scaled coefficient
Gln	Glutamine	1.177	−0.129
PC aa C42:4	Phosphatidylcholine diacyl C42:4	1.168	0.007
lysoPC a C26:1	lysoPhosphatidylcholine acyl C26:1	1.155	0.035
PC ae C40:3	Phosphatidylcholine acly-alkyl C40:3	1.152	0.009
lysoPC a C6:0	lysoPhosphatidylcholine acyl C6:0	1.145	0.044
PC ae C38:1	Phosphatidylcholine acly-alkyl C38:1	1.139	0.035
PC ae C44:3	Phosphatidylcholine acly-alkyl C44:3	1.131	0.024
PC ae C30:2	Phosphatidylcholine acyl-alkyl C 30:2	1.116	−0.015
PC ae C40:4	Phosphatidylcholine acly-alkyl C40:4	1.109	0.009
PC ae C42:1	Phosphatidylcholine acly-alkyl C42:1	1.100	0.029
Ser	Serine	1.097	0.115
PC aa C40:2	Phosphatidylcholine diacyl C40:2	1.095	−0.002
PC aa C40:3	Phosphatidylcholine diacyl C40:3	1.095	0.007
PC ae C40:5	Phosphatidylcholine acly-alkyl C40:5	1.085	0.011
lysoPC a C24:0	lysoPhosphatidylcholine acyl C24:0	1.068	0.042
C18:1	Octadecenoylcarnitine	1.059	0.073
PC ae C38:3	Phosphatidylcholine acly-alkyl C38:3	1.058	−0.009
PC ae C38:2	Phosphatidylcholine acly-alkyl C38:2	1.052	0.004
PC aa C42:2	Phosphatidylcholine diacyl C42:2	1.034	−0.042
PC ae C40:2	Phosphatidylcholine acly-alkyl C40:2	1.033	0.022
C18:2	Octadecadienylcarnitine	1.029	0.114
PC aa C40:1	Phosphatidylcholine diacyl C40:1	0.999	−0.022
PC ae C42:4	Phosphatidylcholine acly-alkyl C42:4	0.998	0.003
PC aa C24:0	Phosphatidylcholine diacyl C24:0	0.987	0.008
Met	Methionine	0.984	−0.028
PC ae C36:1	Phosphatidylcholine acly-alkyl C36:1	0.978	0.002
PC aa C42:5	Phosphatidylcholine diacyl C42:5	0.977	0.011
PC ae C42:2	Phosphatidylcholine acly-alkyl C42:2	0.974	0.004
PC ae C30:1	Phosphatidylcholine acly-alkyl C30:1	0.968	−0.017
C16:1	Hexadecenoyl-L-carnitine	0.962	0.005
PC ae C42:3	Phosphatidylcholine acly-alkyl C42:3	0.957	−0.030
lysoPC a C28:0	lysoPhosphatidylcholine acyl C28:0	0.944	0.033
C3:1	Propenoylcarnitine	0.938	0.031
C16	Hexadecanoylcarnitine	0.927	0.103
PC ae C40:1	Phosphatidylcholine acly-alkyl C40:1	0.922	−0.037
C3-OH	Hydroxypropionylcarnitine	0.913	0.013
lysoPC a C26:0	lysoPhosphatidylcholine acyl C26:0	0.911	0.012
Val	Valine	0.906	−0.063
Orn	Ornithine	0.896	0.201
C16:1-OH	Hydroxyhexadecenoyl-L-carnitine	0.885	0.062
PC aa C26:0	Phosphatidylcholine diacyl C26:0	0.880	0.033
PC ae C42:5	Phosphatidylcholine acly-alkyl C42:5	0.875	−0.001
Trp	Tryptophan	0.841	−0.067
PC ae C38:6	Phosphatidylcholine acly-alkyl C38:6	0.837	−0.112
C10:1	Decenoylcarnitine	0.828	−0.137
C3	Propionylcarnitine	0.827	−0.023
PC ae C36:5	Phosphatidylcholine acly-alkyl C36:5	0.780	−0.094
SM C22:3	Sphingomyeline C22:3	0.708	−0.059

To evaluate whether the identified CRC profile is significantly derived from the stage IVa subgroup of patients, we generated a discriminant model for separation of 5 classes: 4 CRC stages and disease free controls,. This analysis included all samples contained in the training and validation sets (*N* = 100) (Additional file 3: Fig. S2a). The model could strongly distinguish all CRC stages from the control group on the first predictive component (R^2^Y = 0.46 and Q^2^Y = 0.30, *p*-value <0.00001). On the second component, stage IVa patients were distinguished from all locoregional CRC patients. The overlap observed in locoregional CRC stages could be either due to the similarity of their metabolomic profile (relative to their difference with control or stage IVa groups; Additional file 1: Table S2), or it may be secondary to a smaller sample size in each of the three locoregional classes. In all, the data demonstrate that there are stage-associated signatures, but there is also a signature that distinguishes CRC patients as a whole from disease-free controls.

**Fig. 2:**
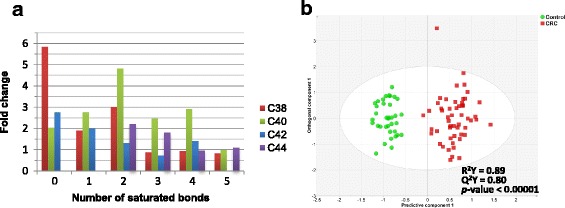
Combined metabolomic profile of colorectal cancer as determined by GC-MS and MS/MS. **a** Fold changes of the compounds of phosphatidylcholine acyl-alkyl class, categorized by the cumulative carbon length of side fatty acids, **b** Supervised (OPLS-DA) scores scatter plot of colorectal cancer and controls

Figure 1b describes the correlation coefficients of the most important metabolites contributing to the CRC metabolomic profile. Glutamine was the most important metabolite in the projection derived from FIA-MS/MS (decreased in CRC), followed by phosphatidylcholine acyl –alkyls C38:1 and C40:3 (increased and decreased in CRC, respectively). In univariate t-test, glutamine was the most differentially abundant metabolite, as well (*p*-value = 2.69e-10). Ornithine and serine had the highest positive correlations with CRC, while decenoylcarnitine (C10:1) had the highest negative correlation with CRC (Fig. 1b). Of 14 amino acids explored, methionine, valine and tryptophan were significantly reduced in CRC, while ornithine and serine had increased levels. The remaining amino acids did not demonstrate any considerable significance in our model. There was no clear association with stage (Fig. 1c and d). Figure 1e and f illustrate the distribution of changes in the PCs in CRC, based on the length of carbon chains and the number of unsaturated bonds. Five lysophosphatidylcholines (Lyso-PC) were particularly elevated in CRC; none was decreased. This has not been previously reported. Phosphatidylcholines are important constituents of cell membranes, as well as mediators in lipid metabolism. Alterations of the phosphatidyl acyl-alkyl compounds with 38 to 44 carbons in CRC were also observed. These perturbations are represented as fold changes per carbon content in Fig. 2a and are discussed later.

To further enhance the capability of this signature biomarker, we evaluated a combinatorial biomarker composed of the 41 compound GC-MS driven signature we previously defined [5] and this 48 metabolite FIA-MS/MS signature. The inclusive model had an R^2^Y score of 0.91 and a Q^2^Y score of 0.84 (CV-ANOVA *p*-value <0.00001). When we regenerated the model with the most informative metabolites (46 compounds, Table 3), the model had an R^2^Y score of 0.89 and a Q^2^Y score of 0.80 with CV-ANOVA *p*-value <0.00001 (Fig. 2b). With permutations consisting of random assignments of samples to diagnostic classes [12], permutated models performed poorly. That is, with 10,000 permutations, the Q^2^Y = −0.11 (compared to 0.84 in our model); and root mean square error of prediction (RMSEP) = 0.56 (compared to 0.11 in our model). In all, the permutated models and our model were significantly different (*P* < 0.00001), and our model had a superior performance than models based on random class assignment. Moreover, in a permutation test consisting of random metabolite combinations in actual diagnostic classes, the Q2-intercept was −0.59, which is a reflection of a model with a high degree of robustness and reliability.

**Table 3:** List of 46 metabolites in combined analysis of targeted MS/MS and GC-MS spectra of colorectal cancer vs. healthy controls. VIP and multivariate correlation coefficients are provided (PC: Phosphatidylcholine, Lyso-PC: lysophosphatidylcholine, ae: acyl-alkyl, aa: diacyl, SM: sphingomyelin)

Metabolite	VIP	Centered and scaled coefficient
Serine	0.65	1.81
Methionine	0.63	1.54
Phosphatidylcholine acly-alkyl C40:3	0.90	1.43
Phosphatidylcholine acly-alkyl C38:1	0.88	1.27
Phosphatidylcholine acly-alkyl C44:3	0.80	1.08
Propenoylcarnitine	0.68	1.04
Phosphatidylcholine acly-alkyl C40:5	0.74	0.84
Phosphatidylcholine diacyl C24:0	0.76	0.83
lysoPhosphatidylcholine acyl C28:0	0.71	0.71
Hydroxyhexadecenoyl-L-carnitine	0.59	0.66
Hexadecanoylcarnitine	0.63	0.63
Octadecadienylcarnitine	0.67	0.60
Phosphatidylcholine acly-alkyl C40:4	0.83	0.57
Phosphatidylcholine diacyl C40:3	0.80	0.50
Phosphatidylcholine diacyl C40:2	0.83	0.47
Phosphatidylcholine acly-alkyl C38:3	0.82	0.47
lysoPhosphatidylcholine acyl C26:1	0.75	0.36
Phosphatidylcholine diacyl C42:5	0.63	0.33
Phosphatidylcholine acly-alkyl C42:1	0.78	0.30
Phosphatidylcholine diacyl C26:0	0.59	0.26
Phosphatidylcholine diacyl C42:4	0.84	0.21
Phosphatidylcholine acly-alkyl C38:6	0.64	0.15
Phosphatidylcholine acly-alkyl C38:2	0.79	0.15
Ethylene glycol, di-TMS	4.85	0.08
lysoPhosphatidylcholine acyl C6:0	0.64	0.06
Propionylcarnitine	0.61	−0.01
lysoPhosphatidylcholine acyl C26:0	0.68	−0.03
Phosphatidylcholine acly-alkyl C40:2	0.71	−0.05
Octadecenoylcarnitine	0.74	−0.06
Phosphatidylcholine acly-alkyl C42:2	0.66	−0.12
Phosphatidylcholine diacyl C40:1	0.69	−0.30
lysoPhosphatidylcholine acyl C24:0	0.73	−0.34
Phosphatidylcholine acly-alkyl C40:1	0.61	−0.46
Phosphatidylcholine acly-alkyl C42:5	0.60	−0.49
Phosphatidylcholine acly-alkyl C36:5	0.64	−0.56
Hydroxypropionylcarnitine	0.66	−0.62
Phosphatidylcholine acly-alkyl C42:4	0.67	−0.63
Valine	0.61	−0.64
Sphingomyeline C22:3	0.60	−0.80
Phosphatidylcholine diacyl C42:2	0.72	−0.89
Phosphatidylcholine acly-alkyl C36:1	0.67	−0.92
Glutamine	0.75	−0.96
Phosphatidylcholine acly-alkyl C42:3	0.63	−0.97
Tryptophan	0.63	−1.00
Phosphatidylcholine acyl-alkyl C 30:2	0.71	−1.12
Hexadecenoyl-L-carnitine	0.68	−1.71

### Detection of very early stage disease

Sporadic colorectal adenocarcinoma is preceded by the formation of adenomatous lesions (polyps) as its precursor. Sera from 31 average-risk adenoma patients and 31 age- and gender- matched healthy controls in the age range of 50 to 70 years old, to evaluate whether metabolomic profiling can also detect this very early stage of the disease. In all cases, one adenoma <1 cm in diameter was detected by colonoscopy, except for one case, that had two adenomas. Clinical factors for these groups are summarized in Table 1.

**Fig. 3:**
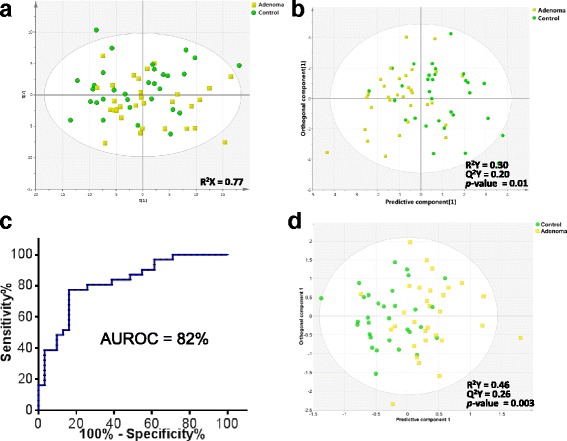
Metabolomic profile of colorectal adenoma as determined by targeted MS/MS. **a** PCA comparison of colorectal adenoma and disease-free controls. **b** Supervised (OPLS-DA) analysis scores scatter plot of adenomas and controls. **c** ROC curve from internal cross-validation, **d** Supervised (OPLS-DA) analysis scores scatter plot of combined metabolomic profile of adenomas and controls as determined by GC-MS and MS/MS

Adenomas and disease-free controls were analyzed on the FIA-MS/MS platform. An unsupervised multivariate analysis, using 146 quantitative concentrations revealed no latent pattern or clustering (8 components, R^2^X = 0.77) (Fig. 3a). A supervised analysis (OPLS-DA) with a focused analysis of 9 metabolites identified by data filtration (*p* < 0.30, Table 4 and Additional file 1: Table S3) provided the basis of a model discriminating adenoma and disease-free controls (R^2^Y = 0.30, Q^2^Y = 0.20, CV-ANOVA *p*-value = 0.01, one orthogonal and one predictive component) (Fig. 3b). The AUROC on internal cross-validation was 82% (95% CI, 0.72–0.93) (Fig. 3c). Therefore, the quantitative analysis using this set of 9 metabolites is comparable to the metabolomic biomarker derived previously by GC-MS, comprised of 17 metabolites [5].

**Table 4:** List of 9 metabolites in targeted MS/MS analysis of colorectal adenoma vs. healthy controls. VIP and multivariate correlation coefficients are provided (PC: Phosphatidylcholine, Lyso-PC: lysophosphatidylcholine, ae: acyl-alkyl, aa: diacyl, SM: sphingomyelin)

Abbreviated Name	Metabolite	VIP	Centered and scaled coefficient
Trp	Tryptophan	1.12	0.15
C14:2	Tetradecadienylcarnitine	1.12	−0.09
C12:1	Dodecenoylcarnitine	1.07	−0.07
C16:2	Hexadecadienylcarnitine	1.05	−0.05
C10:1	Decenoylcarnitine	1.01	−0.07
Pro	Proline	0.91	0.20
C14:1-OH	Hydroxytetradecenoylcarnitine	0.91	−0.08
PC ae C40:2	Phosphatidylcholine acly-alkyl C40:2	0.90	0.18
lysoPC a C17:0	lysoPhosphatidylcholine acyl C17:0	0.86	0.16

Finally, we sought to determine whether the adenoma profile identified by the quantitative approach could be combined with the adenoma profile defined by GC-MS [5] to enhance the performance of the biomarker. The combined signature composed of 14 GC-MS metabolites and 9 quantitative metabolites could generate a model which was slightly better than the quantitative signature alone (R^2^Y = 0.46, Q^2^Y = 0.26, CV-ANOVA *p*-value = 0.003, Q2-intercept = −0.31). Selecting for the most important metabolites, we generated a biomarker pattern consisting of 18 compounds (R^2^Y = 0.33, Q^2^Y = 0.21, CV-ANOVA *p*-value = 0.01, Q2-intercept = −0.26) (Fig. 3d and Table 5). Overall, the combinatorial signature had a better performance in the training study.

**Table 5:** List of 18 metabolites in combined analysis of targeted MS/MS and GC-MS spectra of colorectal cancer vs. healthy controls. VIP and multivariate correlation coefficients are provided (PC: Phosphatidylcholine, Lyso-PC: lysophosphatidylcholine, ae: acyl-alkyl, aa: diacyl, SM: sphingomyelin)

Metabolite	VIP	Centered and scaled coefficient
C14:2 (Tetradecadienyl carnitine)	1.14	0.09
C12:1 (Dodecenoylcarnitine)	1.13	−0.18
Unidentified_compound (RI = 1448.35)	1.11	0.40
Unidentified_compound (RI = 2495.71)	1.11	0.20
Proline	1.11	−0.22
C10:1 (Decenoylcarnitine)	1.10	−0.06
Unidentified_compound (RI = 2378.86)	1.10	0.39
Cystine (4TMS)	1.10	−0.30
C16:2 (Hexadecadienylcarnitine)	1.09	0.07
C14:1-OH (Hydroxytetradecenoylcarnitine)	1.08	−0.24
Glyceric acid (3TMS)	1.05	0.35
Unidentified_compound (RI = 1416.54)	0.98	0.39
Glutamic acid (3TMS)	0.96	−0.19
Unidentified_compound (RI = 2933.74)	0.81	0.26
Unidentified_compound (RI = 1977.64)	0.79	−0.33
lysoPC a C17:0	0.74	0.31
Heptadecanoic acid (1TMS)	0.70	0.20
Unidentified_compound (RI = 1101.40)	0.67	−0.40

## Discussion

CRC is often diagnosed at later stages when prognosis is worse and treatment algorithms become more complex and expensive. Early detection of CRC is, therefore, desirable. Given the high incidence of CRC, substantial efforts have been made to devise cost-effective screening strategies that can be applied to the general population. Presently, such strategies employ direct examination of the colon by colonoscopy in an enriched population; the enriched population is typically identified by symptoms or by positive fecal occult blood tests. However, fecal tests are hampered by low compliance as well as low sensitivity. A blood test may provide a more convenient and acceptable alternative, which may enhance compliance. This may make it more practicable to perform on a regular (e.g. yearly) basis, potentially reducing missed diagnoses. With this in mind, our group has sought to develop a metabolomics-based blood test that can be used for early diagnosis of colorectal neoplasia. Previously, we demonstrated the power of this approach using a semi-quantitative analytical platform, GC-TOF-MS. Here, we sought to determine whether a more comprehensive metabolomic signature could enhance the performance of the diagnostic biomarker. Indeed, an extension of the metabolite coverage to include additional classes of metabolites does strengthen the performance of the biomarker pattern. We also sought to explore how a fully quantitative metabolomic assay might perform if used to identify a specific disease state. There was a marked distinction in the metabolomic profiles of CRC and controls, using this quantitative approach.

Ultimately, any diagnostic assay must be repeatable and accurate. Most metabolomics efforts involve the use of semi-quantitative approaches where individual metabolites are compared between disease states without specifically quantifying those metabolites. The measurement variability of mass spectrometry-based approaches can also be substantial. This can be problematic given that disease-specific perturbations in individual metabolites may be small. Therefore, measurement errors may contribute to overlap of distributions, which will affect the accuracy of disease classification. As we applied a comprehensive quantitative approach, we observed that the measurement variance did not change much. However, the certainty of the quantitative measurement of individual metabolites was enhanced because the inclusion of isotope-labelled standards in the Biocrates kit minimizes the variability arising from non-biological (analytical) sources. This directly affected the degree of distinction between disease states, reducing diagnostic overlap. Therefore, it is clear that, as metabolomic biomarkers are identified and validated, it will be essential to develop assays with well-selected internal controls as the tests are developed further for clinical practice.

Using FIA-MS/MS, we have significantly broadened coverage of the metabolome in comparison to our previous studies using GC-TOF-MS and 1H–NMR spectroscopy, which enabled identification of carbohydrates, some amino acids, and simple fatty acid (FA) structures [4, 5]. The quantitative MS/MS assay used in the present study included phosphatidylcholines, sphingomyelins, acylcarnitines, and amino acids. When a biomarker pattern was formulated based on both GC-TOF-MS and FIA-MS/MS, the diagnostic accuracy improved. We believe that that is mostly because the specificity was enhanced by the more comprehensive metabolomic biomarker. It is possible that the enhanced performance of the combined model was a function of overfitting. However, as these metabolites were shortlisted from a larger list of already informative metabolites, and since metabolites from each platform contributed to the combined biomarker, we believe that this is not a major factor in the encouraging performance of the combined platform biomarker pattern. As we embark on further validation studies, there may be continued refinement of the optimal diagnostic biomarkers. In particular, as we test external validation cohorts, it will be important to optimize the metabolomic biomarkers diagnostic for colorectal adenoma and carcinoma.

The identification of diagnostic metabolites on FIA-MS/MS in addition to the metabolites previously described from GC-MS studies is quite instructive as we continue development of an assay ready for the clinic. Going forward, we will create a quantitative assay based on GC-MS/MS, which will allow us to measure all of the metabolites detected in GC-TOF-MS and FIA-MS/MS. Following this, the biomarker can be optimized regarding test performance characteristics, limiting measurement to the most consistently informative compounds. The additional advantage of using GC-MS/MS is that the extra dimension of fractionation will provide more specific identification of individual compounds.

There are few studies describing changes in circulating lipids in CRC [21]. The data generated in this study provide unique observations that require additional investigation. A number of PCs are increased in CRC. HDL transfers circulating PCs, and the fact that HDL-C levels are increased in CRC is consistent with this observation [7]. PCs with 40 carbons are especially increased. The relative accumulation of PCs with 40 carbons might point to dysregulated mechanisms of fatty acid elongation. Interestingly, it has been shown that some germline single nucleotide polymorphisms of fatty acid desaturases FADS1 and FADS2 are associated with a higher risk of CRC [22]; and FADS1 and FADS2 are upregulated in CRC [22]. FADS1 and FADS2 mediate the conversion of linoleic acid (18:2n-6) to arachidonic acid (20:4n-6) [23], a precursor to polyunsaturated fatty acids with longer than 20 carbons. It is possible that this increased abundance of fatty acids varying in length from 22 to 24 carbons, which comprise the components of PCs containing 40 carbons, is responsible for the particularly high levels of PCs with 40 carbons. There is also a report on a large number of CRC patients found to have low levels of hydroxylated polyunsaturated ultra-long chain fatty acid metabolites (PUFAs) [24]. The biological mechanism for this is unknown. However, it is now known that the levels of PUFAs do not correlate with disease burden, and it appears that reduction in PUFAs is actually more reflective of risk of CRC [25]. This is important because it will be essential to evaluate whether any metabolomics-based biomarker of CRC is proportional to disease burden, and whether it disappears with resection.

LysoPCs, products of PC breakdown, do not change much in comparison (Table 2). One intriguing observation is that LysoPC C28:0 increases and LysoPC 28:1 decreases; LysoPC 26:1 increases and Lyso 26:0 decreases. This imbalance suggests that there is some alteration in the mechanisms involved in fatty acid saturation or elongation that accompanies CRC. Li et al. have also reported alterations in FTICR-MS measurements of 4 LysoPCs with shorter chains, including poly-unsaturated LysoPCs with 16, 18, 20, and 22 carbons along with 11 other compounds, mainly free fatty acids including palmitic amide, oleamide, hexadecanedioic acid, octadecanoic acid, eicosatrienoic acid [26]. Although we did not observe these LysoPCs to be changed in our cohort, the acylcarnitines of the fatty acids were significantly altered (Additional file 1: Table S1).

Given that CRC is associated with a higher cell turnover, it might be expected that CRC would be associated with higher levels of PC-diacyls, a normal component of cell membranes. However, it is unclear why there was an even more pronounced increase in levels of PC-acyl-alkyls in CRC, which are not major cell membrane constituents. It is also not clear which changes in circulating lipid products are secondary to host-derived changes in fat metabolism and nutrition, and which perturbations are secondary to tumor metabolism. This will require further study.

A number of groups have similarly characterized the metabolomic features of the colorectal cancer (CRC) using various analytical platforms ([21, 27–30]). These studies are very difficult to compare, because of the diversity of the analytical platforms, differences in compared groups, and differences in genetic and environmental factors. The present work is unique in that it involves quantifying circulating lipids in very early disease (adenoma) in addition to invasive CRC. Admittedly, this was a relatively small sample size and only the invasive CRC signature was externally validated. Therefore, additional validation will be required. In addition, it will be imperative to test any signature in diverse clinical cohorts to ensure that any environmental and genetic factors do not significantly contribute to the diagnostic metabolomic profile.

In summary, we have utilized a quantitative metabolomic assay to determine levels of various circulating metabolites, including lipid metabolites. The biomarker pattern comprised of metabolites that are most significantly altered clearly distinguishes CRC and adenoma from normal controls. The fact that adenomas are detectable in blood suggests that a metabolomic test could be adapted for use as a screening tool. The performance characteristics of the biomarker profile are quite encouraging, especially when combined with the GC-MS biomarker signature previously described. It will be important going forward to devise a single assay that allows quantitation of all significant classifier metabolites, to optimize the diagnostic performance of any future blood test. It will also be essential to confirm that the metabolomic signal is distinguished with treatment. In addition to providing direction on the development of a diagnostic assay, we have reported unique observations that will lead to future investigations related to how tumor and host metabolism are altered in CRC, and how those separate processes interact. Following up with this development, we will be testing a population-based cohort to assess the clinical performance and effectiveness of these metabolomic profiles.

## Conclusions

This study denotes promising quantitative metabolomic signatures for the identification of CRC and colorectal adenoma, as well as an extensive description of a number of perturbations in the metabolism of phosphatidylcholines in CRC. Future work will involve creation of an assay based on these signatures, as well as further validation experiments.

## Additional files

Additional file 1: Fig. S1. A. Principal Component Analysis (PCA) of CRC and control samples in the training set. B. PCA of CRC and control samples in the training set, colored by pre-sampling chemotherapy status. C. PCA of CRC samples from 4 stages, colored by their TNM stages. D. PCA of CRC samples from 4 stages, colored by pre-sampling chemotherapy status. E. PCA of CRC stage IVa patients, to study the potential confounding effect of chemotherapy on the described CRC metabolomic profile. (DOCX 103 kb)

Additional file 2: Fig. S2. Metabolomic profile of CRC, Stages I to IVa by OPLS-DA supervised analysis. A. Scores scatter plot of discriminant analysis. Model characteristics are indicated. The first component clearly distinguishes between CRC and control groups, while the second component identifies locoregional CRC from liver-metastatic CRC (stage IVa). (PPTX 8816 kb)

Additional file 3: Tables S1. **S2** and **S3.** Lists of metabolites incorporated into each metabolomic signature for colorectal cancer and colorectal adenoma (PPTX 2288 kb)

## Abbreviations

CR: Colorectal
CRC: Colorectal cancer
CV-ANOVA: Cross validation-analysis of variance
FA: Fatty acid
FIA: Flow injection analysis
GC-MS: Gas chromatography-mass spectrometry
GC-ToF-MS: Gas chromatography-time of flight-mass spectrometry
Lyso-PC: Lyso phosphatidylcholine
MRM: Multiple reaction monitoring
MS: Mass spectrometry
MS/MS: Tandem mass spectrometry
NMR: Nuclear magnetic resonance spectroscopy
OPLS-DA: Orthogonal partial least scores- discriminant analysis
PC: Phosphatidylcholine
PCA: Principal component analysis
PLS: Partial least scores analysis
RMSEP: Root mean square error of prediction
