# Supplementary material for: A quantitative multimodal metabolomic assay for colorectal cancer
Source: BMC Cancer. 2018 Jan 4;18:26. doi: 10.1186/s12885-017-3923-z (PMC5755335; doi:10.1186/s12885-017-3923-z)
Supplement: Supplementary file 1 — A. Principal Component Analysis (PCA) of CRC and control samples in the training set. B. PCA of CRC and control samples in the training set, colored by pre-sampling chemotherapy status. C. PCA of CRC samples from 4 stages, colored by their TNM stages. D. PCA of CRC samples from 4 stages, colored by pre-sampling chemotherapy status. E. PCA of CRC stage IVa patients, to study the potential confounding effect of chemotherapy on the described CRC metabolomic profile. (DOCX 103 kb) [file 12885_2017_3923_MOESM1_ESM.docx]

**Supplementary Tables**

*Supplementary Table 1*. List of 146 metabolites with Welch’s inter-group *p*-values for the whole set and only training set in targeted MS/MS analysis of colorectal cancer vs. healthy controls. Mean and median serum levels are also provided. The unit is micromolar (µM) (PC: Phosphatidylcholine, Lyso-PC: lysophosphatidylcholine, ae: acyl-alkyl, aa: diacyl, SM: sphingomyelin).

*Supplementary Table 2.* List of 40 metabolites with Kruskal-Wallis inter-group *p*-value < 0.30 in targeted MS/MS analysis of locoregional colorectal cancer (PC: Phosphatidylcholine, Lyso-PC: lysophosphatidylcholine, ae: acyl-alkyl, aa: diacyl, SM: sphingomyelin)

*Supplementary Table 3.* List of 75 metabolites with Welch’s inter-group *p*-value < 0.30 in the targeted analysis of colorectal adenoma vs. matched controls; red indicates relative increase and green indicates a relative decrease. (PC: Phosphatidylcholine, Lyso-PC: lysophosphatidylcholine, ae: acyl-alkyl, aa: diacyl, SM: sphingomyelin)

Supplementary Table 1. List of 146 metabolites with Welch’s inter-group *p*-values for the whole set and only training set in targeted MS/MS analysis of colorectal cancer vs. healthy controls. Mean and median serum levels are also provided. The unit is micromolar (µM) (PC: Phosphatidylcholine, Lyso-PC: lysophosphatidylcholine, ae: acyl-alkyl, aa: diacyl, SM: sphingomyelin).

| **ID** | **Metabolite** | **Training *p*-value** | **Whole-set *p*-value** | **Fold Change** | **Control Mean(SD)** | **Control Median** | **CRC Mean(SD)** | **CRC Median** |
| --- | --- | --- | --- | --- | --- | --- | --- | --- |
| **Gln** | Glutamine | 2.69E-10 | 1.05E-17 | 0.63826 | 821.2 (99.04) | 798 | 524.2 (184.2) | 516.42 |
| **PC ae C40:3** | Phosphatidylcholine acly-alkyl C40:3 | 3.68E-08 | 5.21E-14 | 4.823938 | 1.209 (0.488) | 1.15 | 5.834 (3.718) | 4.78 |
| **PC ae C38:1** | Phosphatidylcholine acly-alkyl C38:1 | 1.05E-07 | 1.11E-13 | 5.841619 | 0.751 (0.495) | 0.701 | 4.386 (2.981) | 3.515 |
| **PC ae C40:4** | Phosphatidylcholine acly-alkyl C40:4 | 1.77E-07 | 4.39E-13 | 2.471607 | 2.563 (0.709) | 2.51 | 6.335 (3.199) | 5.805 |
| **PC ae C42:1** | Phosphatidylcholine acly-alkyl C42:1 | 1.17E-06 | 3.44E-11 | 2.766367 | 0.444 (0.132) | 0.415 | 1.229 (0.756) | 1.078 |
| **PC ae C38:3** | Phosphatidylcholine acly-alkyl C38:3 | 1.56E-06 | 1.12E-11 | 3.002224 | 5.012 (1.352) | 4.88 | 13.864 (8.26) | 12.55 |
| **PC aa C42:4** | Phosphatidylcholine diacyl C42:4 | 2.64E-06 | 3.21E-11 | 3.169053 | 0.211 (0.071) | 0.199 | 0.669 (0.441) | 0.572 |
| **PC ae C40:2** | Phosphatidylcholine acly-alkyl C40:2 | 2.69E-06 | 1.55E-10 | 2.767204 | 2.109 (0.661) | 2.02 | 3.977 (1.822) | 3.579 |
| **PC ae C38:2** | Phosphatidylcholine acly-alkyl C38:2 | 2.82E-06 | 2.55E-11 | 1.887948 | 2.489 (0.799) | 2.36 | 7.473 (4.762) | 6.4 |
| **PC ae C40:5** | Phosphatidylcholine acly-alkyl C40:5 | 3.40E-06 | 8.01E-11 | 2.906029 | 4.997 (1.445) | 4.62 | 9.434 (4.273) | 8.875 |
| **PC aa C40:2** | Phosphatidylcholine diacyl C40:2 | 5.15E-06 | 1.17E-10 | 4.192398 | 0.379 (0.143) | 0.367 | 1.588 (1.216) | 1.275 |
| **PC aa C40:3** | Phosphatidylcholine diacyl C40:3 | 5.31E-06 | 8.93E-11 | 1.8855 | 0.651 (0.204) | 0.626 | 1.893 (1.233) | 1.535 |
| **C18:2** | Octadecadienyl carnitine | 5.43E-06 | 2.53E-09 | 1.722362 | 0.077 (0.024) | 0.076 | 0.128 (0.053) | 0.118 |
| **C18:1** | Octadecenoyl carnitine | 6.98E-06 | 7.70E-10 | 3.011287 | 0.201 (0.056) | 0.194 | 0.346 (0.148) | 0.326 |
| **C16** | Hexadecanoyl carnitine | 9.14E-06 | 3.29E-08 | 1.665684 | 0.135 (0.031) | 0.131 | 0.2 (0.074) | 0.184 |
| **PC ae C44:3** | Phosphatidylcholine acly-alkyl C44:3 | 1.46E-05 | 8.76E-10 | 2.208677 | 0.134 (0.056) | 0.126 | 0.403 (0.287) | 0.368 |
| **C3:1** | Propenoyl carnitine | 1.74E-05 | 4.59E-08 | 1.773936 | 0.007 (0.002) | 0.007 | 0.018 (0.013) | 0.008 |
| **PC ae C36:1** | Phosphatidylcholine acly-alkyl C36:1 | 2.29E-05 | 6.23E-09 | 1.596352 | 10.434 (2.72) | 10.2 | 18.509 (9.04) | 17.163 |
| **C3-OH** | Hydroxypropionyl carnitine | 3.67E-05 | 7.62E-07 | 1.548894 | 0.012 (0.004) | 0.011 | 0.029 (0.025) | 0.014 |
| **C16:1** | Hexadecenoyl-L- carnitine | 3.89E-05 | 1.53E-08 | 2.302437 | 0.043 (0.011) | 0.042 | 0.067 (0.026) | 0.063 |
| **PC ae C42:4** | Phosphatidylcholine acly-alkyl C42:4 | 4.18E-05 | 6.40E-09 | 0.727134 | 1.125 (0.292) | 1.07 | 1.795 (0.729) | 1.778 |
| **Met** | Methionine | 5.82E-05 | 3.14E-08 | 1.477889 | 38.483 (5.61) | 38.4 | 27.98 (11.69) | 28.1 |
| **lysoPC a C6:0** | lysoPhosphatidylcholine acyl C6:0 | 6.40E-05 | 8.06E-08 | 1.376478 | 0.021 (0.006) | 0.021 | 0.037 (0.014) | 0.035 |
| **Ser** | Serine | 7.14E-05 | 3.31E-08 | 2.499728 | 149.73 (21.4) | 146 | 206.1 (66.83) | 204 |
| **PC aa C42:2** | Phosphatidylcholine diacyl C42:2 | 7.78E-05 | 3.35E-09 | 1.832346 | 0.295 (0.083) | 0.282 | 0.652 (0.398) | 0.545 |
| **PC aa C24:0** | Phosphatidylcholine diacyl C24:0 | 0.0001 | 2.86E-08 | 1.958051 | 0.132 (0.045) | 0.128 | 0.304 (0.208) | 0.246 |
| **C16:1-OH** | Hydroxyhexadecenoyl-L-carnitine | 0.0001 | 8.62E-07 | 1.776487 | 0.014 (0.003) | 0.013 | 0.018 (0.006) | 0.017 |
| **lysoPC a C28:0** | lysoPhosphatidylcholine acyl C28:0 | 0.0002 | 5.45E-07 | 1.626873 | 0.392 (0.117) | 0.352 | 0.782 (0.533) | 0.607 |
| **PC ae C42:2** | Phosphatidylcholine acly-alkyl C42:2 | 0.0002 | 5.07E-08 | 1.994523 | 0.754 (0.22) | 0.764 | 1.382 (0.763) | 1.275 |
| **PC aa C40:1** | Phosphatidylcholine diacyl C40:1 | 0.0003 | 7.46E-08 | 2.513331 | 0.481 (0.134) | 0.468 | 0.941 (0.576) | 0.812 |
| **C10:1** | Decenoylcarnitine | 0.0003 | 0.0012 | 1.340532 | 0.28 (0.079) | 0.265 | 0.221 (0.097) | 0.196 |
| **lysoPC a C26:0** | lysoPhosphatidylcholine acyl C26:0 | 0.0003 | 3.77E-06 | 0.794266 | 0.461 (0.173) | 0.438 | 0.935 (0.707) | 0.748 |
| **Orn** | Ornithine | 0.0004 | 0.0007 | 1.615831 | 103.29 (32.5) | 94.9 | 131.0 (47.37) | 129.343 |
| **PC aa C42:5** | Phosphatidylcholine diacyl C42:5 | 0.0005 | 4.86E-07 | 1.618758 | 0.463 (0.147) | 0.474 | 0.753 (0.374) | 0.696 |
| **PC ae C42:5** | Phosphatidylcholine acly-alkyl C42:5 | 0.0005 | 2.48E-06 | 1.410425 | 2.73 (0.738) | 2.64 | 3.85 (1.489) | 3.597 |
| **PC ae C40:1** | Phosphatidylcholine acly-alkyl C40:1 | 0.0005 | 1.48E-06 | 2.027523 | 1.756 (0.473) | 1.8 | 2.843 (1.519) | 2.6 |
| **PC ae C42:3** | Phosphatidylcholine acly-alkyl C42:3 | 0.0010 | 1.37E-06 | 1.322905 | 1.028 (0.301) | 0.973 | 1.661 (0.872) | 1.565 |
| **C18** | Octadecanoyl carnitine | 0.0011 | 0.0002 | 1.600445 | 0.062 (0.014) | 0.06 | 0.078 (0.027) | 0.075 |
| **PC aa C42:1** | Phosphatidylcholine diacyl C42:1 | 0.0016 | 6.38E-06 | 1.815504 | 0.37 (0.133) | 0.354 | 0.593 (0.322) | 0.508 |
| **lysoPC a C28:1** | lysoPhosphatidylcholine acyl C28:1 | 0.0029 | 2.69E-05 | 0.654887 | 0.585 (0.153) | 0.566 | 1.059 (0.8) | 0.824 |
| **PC ae C44:4** | Phosphatidylcholine acly-alkyl C44:4 | 0.0029 | 6.62E-05 | 1.810918 | 0.454 (0.099) | 0.445 | 0.599 (0.239) | 0.542 |
| **PC aa C26:0** | Phosphatidylcholine  diacyl C26:0 | 0.0031 | 0.0008 | 1.307712 | 1.022 (0.234) | 0.972 | 1.309 (0.434) | 1.185 |
| **lysoPC a C26:1** | lysoPhosphatidylcholine acyl C26:1 | 0.0031 | 7.20E-05 | 1.322761 | 0.223 (0.093) | 0.195 | 0.492 (0.36) | 0.378 |
| **xLeu** | Leucine/Isoleucine | 0.0036 | 0.0003 | 1.317752 | 225.15 (42.6) | 217 | 272.19 (82.7) | 271.794 |
| **PC aa C38:1** | Phosphatidylcholine diacyl C38:1 | 0.0039 | 1.50E-05 | 2.203955 | 1.336 (0.624) | 1.2 | 2.426 (1.683) | 1.97 |
| **Gly** | Glycine | 0.0040 | 0.0006 | 1.492496 | 339.76 (93.4) | 327 | 421.5 (138.9) | 426.412 |
| **PC ae C42:0** | Phosphatidylcholine acly-alkyl C42:0 | 0.0041 | 4.03E-06 | 1.775614 | 0.657 (0.165) | 0.617 | 0.869 (0.272) | 0.828 |
| **PC aa C34:1** | Phosphatidylcholine diacyl C34:1 | 0.0045 | 0.0015 | 1.261561 | 252.44 (58.0) | 245 | 303.48 (98.5) | 275.118 |
| **lysoPC a C24:0** | lysoPhosphatidylcholine acyl C24:0 | 0.0050 | 7.57E-05 | 2.115326 | 0.264 (0.081) | 0.245 | 0.469 (0.274) | 0.399 |
| **Val** | Valine | 0.0062 | 0.0008 | 1.389358 | 316.1 (62.34) | 305 | 261.14 (97.6) | 252.778 |
| **PC ae C36:0** | Phosphatidylcholine acly-alkyl C36:0 | 0.0062 | 0.0007 | 1.208947 | 0.97 (0.255) | 0.984 | 1.235 (0.501) | 1.175 |
| **PC aa C40:4** | Phosphatidylcholine diacyl C40:4 | 0.0064 | 0.0021 | 1.208216 | 4.203 (1.25) | 4.13 | 5.264 (2.127) | 5.02 |
| **C3** | Propionylcarnitine | 0.0070 | 1.92E-05 | 0.702606 | 0.466 (0.185) | 0.421 | 0.305 (0.152) | 0.260 |
| **PC ae C38:0** | Phosphatidylcholine acly-alkyl C38:0 | 0.0070 | 7.26E-05 | 0.767237 | 3.44 (1.37) | 3.35 | 5.134 (2.704) | 5.035 |
| **C16-OH** | Hydroxyhexadecanoyl-L-carnitine | 0.0082 | 4.34E-05 | 0.709144 | 0.008 (0.001) | 0.008 | 0.01 (0.003) | 0.01 |
| **C5-DC (C6-OH)** | Glutaryl-L-carnitine / Hydroxyhexanoyl-L-carnitine | 0.0089 | 5.48E-05 | 0.740681 | 0.026 (0.005) | 0.025 | 0.034 (0.014) | 0.0335 |
| **PC ae C30:1** | Phosphatidylcholine acly-alkyl C30:1 | 0.0103 | 0.0002 | 1.240711 | 0.132 (0.076) | 0.139 | 0.28 (0.208) | 0.212 |
| **C5** | Valerylcarnitine | 0.0123 | 0.0004 | 1.273087 | 0.17 (0.053) | 0.16 | 0.13 (0.052) | 0.126 |
| **Trp** | Tryptophan | 0.0124 | 1.15E-06 | 1.268357 | 96.785 (15.0) | 99.6 | 76.87 (23.62) | 76.186 |
| **C4** | Butyrylcarnitine | 0.0133 | 0.0003 | 1.280036 | 0.267 (0.096) | 0.242 | 0.188 (0.117) | 0.16 |
| **PC ae C38:6** | Phosphatidylcholine acly-alkyl C38:6 | 0.0174 | 0.0013 | 0.826085 | 10.911 (4.01) | 9.8 | 8.324 (3.47) | 7.52 |
| **C10:2** | Decadienylcarnitine | 0.0178 | 0.0485 | 2.257804 | 0.052 (0.014) | 0.049 | 0.046 (0.02) | 0.0397 |
| **PC aa C32:2** | Phosphatidylcholine diacyl C32:2 | 0.0179 | 0.0005 | 0.789097 | 5.396 (1.898) | 5 | 3.827 (2.434) | 3.14 |
| **C12:1** | Dodecenoylcarnitine | 0.0187 | 0.0664 | 0.762884 | 0.256 (0.056) | 0.244 | 0.23 (0.088) | 0.207 |
| **Phe** | Phenylalanine | 0.0188 | 0.0003 | 1.202176 | 78.87 (12.05) | 79.6 | 95.29 (30.75) | 90.822 |
| **PC ae C36:5** | Phosphatidylcholine acly-alkyl C36:5 | 0.0188 | 0.0006 | 0.770079 | 16.406 (6.02) | 15.3 | 12.152 (5.51) | 11.152 |
| **PC ae C30:2** | Phosphatidylcholine acyl-alkyl C 30:2 | 0.0200 | 0.0010 | 1.252556 | 0.148 (0.041) | 0.137 | 0.335 (0.315) | 0.247 |
| **C16:2** | Hexadecadienyl carnitine | 0.0231 | 0.0003 | 1.181585 | 0.011 (0.004) | 0.01 | 0.015 (0.007) | 0.014 |
| **C14** | Tetradecanoyl carnitine | 0.0239 | 0.0024 | 1.171618 | 0.055 (0.011) | 0.054 | 0.065 (0.021) | 0.062 |
| **C5-M-DC** | Methylglutaryl carnitine | 0.0257 | 0.0030 | 1.219502 | 0.032 (0.005) | 0.031 | 0.037 (0.01) | 0.035 |
| **PC aa C30:2** | Phosphatidylcholine diacyl C30:2 | 0.0262 | 0.0084 | 0.696587 | 0.099 (0.086) | 0.097 | 0.182 (0.163) | 0.15 |
| **C5:1-DC** | Glutaconyl carnitine | 0.0264 | 0.0035 | 0.825592 | 0.017 (0.004) | 0.016 | 0.02 (0.008) | 0.019 |
| **SM C26:0** | Sphingomyeline C26:0 | 0.0293 | 0.0016 | 1.285134 | 0.205 (0.066) | 0.19 | 0.158 (0.079) | 0.148 |
| **lysoPC a C16:0** | lysoPhosphatidylcholine acyl C16:0 | 0.0305 | 0.0379 | 0.790088 | 132.0 (32.23) | 124 | 153.0 (67.14) | 142.001 |
| **Thr** | Threonine | 0.0314 | 0.0912 | 1.826379 | 117.61 (22.2) | 118 | 128.4 (41.35) | 129 |
| **SM C22:3** | Sphingomyeline C22:3 | 0.0376 | 0.0047 | 0.780678 | 1.81 (0.768) | 1.65 | 1.261 (1.142) | 1.165 |
| **C10** | Decanoyl carnitine | 0.0503 | 0.0318 | 1.168496 | 0.383 (0.184) | 0.33 | 0.303 (0.171) | 0.257 |
| **PC ae C34:0** | Phosphatidylcholine acly-alkyl C34:0 | 0.0567 | 0.1079 | 1.233614 | 1.721 (0.485) | 1.67 | 1.95 (0.93) | 1.75 |
| **C8** | Octanoyl carnitine | 0.0568 | 0.0289 | 0.820303 | 0.266 (0.103) | 0.229 | 0.219 (0.101) | 0.186 |
| **PC aa C34:2** | Phosphatidylcholine diacyl C34:2 | 0.0613 | 0.0349 | 1.11482 | 398.07 (63.4) | 397 | 442.5 (141.8) | 423.583 |
| **C3-DC (C4-OH)** | Malonyl-L-carnitine / Hydroxybutyryl-L-carnitine | 0.0621 | 0.0335 | 0.826305 | 0.065 (0.037) | 0.053 | 0.083 (0.047) | 0.070 |
| **lysoPC a C17:0** | lysoPhosphatidylcholine acyl C17:0 | 0.0646 | 0.2975 | 0.79164 | 2.626 (0.948) | 2.43 | 2.883 (1.518) | 2.452 |
| **SM C18:0** | Sphingomyeline C18:0 | 0.0668 | 0.0335 | 1.152777 | 26.89 (6.696) | 26.6 | 31.00 (12.37) | 29.55 |
| **PC aa C40:6** | Phosphatidylcholine diacyl C40:6 | 0.0729 | 0.0111 | 1.278065 | 39.48 (17.75) | 38.7 | 30.82 (13.86) | 28.786 |
| **PC aa C32:0** | Phosphatidylcholine diacyl C32:0 | 0.0776 | 0.0567 | 1.111625 | 18.344 (4.87) | 17.5 | 21.10 (9.41) | 19.094 |
| **PC aa C34:3** | Phosphatidylcholine diacyl C34:3 | 0.0857 | 0.0440 | 1.159442 | 24.74 (7.28) | 23.9 | 28.78 (12.6) | 27.781 |
| **PC ae C38:4** | Phosphatidylcholine acly-alkyl C38:4 | 0.0903 | 0.1491 | 0.8762 | 16.086 (4.58) | 15.4 | 17.651 (6.23) | 16.525 |
| **PC ae C34:1** | Phosphatidylcholine acly-alkyl C34:1 | 0.0943 | 0.1005 | 0.837061 | 12.168 (2.92) | 12.3 | 13.504 (5.16) | 12.2 |
| **SM C20:2** | Sphingomyeline C20:2 | 0.0978 | 0.0459 | 0.836896 | 0.734 (0.21) | 0.747 | 0.843 (0.33) | 0.804 |
| **lysoPC a C18:0** | lysoPhosphatidylcholine acyl C18:0 | 0.1042 | 0.2089 | 1.163462 | 42.90 (12.40) | 39.7 | 47.02 (20.36) | 45.3 |
| **C18:1-OH** | Hydroxyoctadecenoyl-L-carnitine | 0.1191 | 0.0062 | 1.147939 | 0.009 (0.003) | 0.009 | 0.012 (0.005) | 0.0115 |
| **PC ae C36:4** | Phosphatidylcholine acly-alkyl C36:4 | 0.1211 | 0.0052 | 0.923164 | 22.19 (6.44) | 20.5 | 18.321 (6.93) | 17.7 |
| **C16:2-OH** | Hydroxyhexadecadienyl-L-carnitine | 0.1220 | 0.0114 | 0.869028 | 0.012 (0.003) | 0.011 | 0.014 (0.004) | 0.014 |
| **lysoPC a C14:0** | lysoPhosphatidylcholine acyl C14:0 | 0.1289 | 0.6079 | 0.880018 | 4.098 (0.791) | 3.89 | 4.242 (1.515) | 3.855 |
| **C5-OH (C3-DC-M)** | Methylmalonyl-L-carnitine / Hydroxyvaleryl-L-carnitine | 0.1393 | 0.0464 | 1.150343 | 0.064 (0.009) | 0.065 | 0.059 (0.012) | 0.060 |
| **PC aa C38:6** | Phosphatidylcholine diacyl C38:6 | 0.1432 | 0.0434 | 1.244074 | 113.87 (46.1) | 112 | 95.3 (41.914) | 87.691 |
| **PC ae C34:3** | Phosphatidylcholine acly-alkyl C34:3 | 0.1475 | 0.0160 | 0.896562 | 9.845 (3.528) | 9.15 | 8.076 (3.555) | 7.5 |
| **Pro** | Proline | 0.1530 | 0.1160 | 1.0921 | 210.46 (48.6) | 216 | 233.25 (94.8) | 214.5 |
| **lysoPC a C20:4** | lysoPhosphatidylcholine acyl C20:4 | 0.1544 | 0.4998 | 1.109792 | 8.79 (3.284) | 8.69 | 9.309 (4.409) | 8.968 |
| **C0** | Carnitine | 0.1637 | 0.8055 | 1.133517 | 43.156 (9.01) | 42.1 | 42.61 (13.13) | 41.267 |
| **lysoPC a C20:3** | lysoPhosphatidylcholine acyl C20:3 | 0.1771 | 0.7057 | 1.184703 | 3.013 (1.058) | 2.88 | 3.109 (1.468) | 2.92 |
| **PC ae C36:3** | Phosphatidylcholine acly-alkyl C36:3 | 0.1816 | 0.0401 | 1.108258 | 10.48 (2.804) | 9.82 | 9.182 (3.422) | 8.78 |
| **Arg** | Arginine | 0.1819 | 0.0248 | 0.889935 | 145.44 (20.6) | 146 | 162.14 (51.1) | 154 |
| **PC aa C42:0** | Phosphatidylcholine diacyl C42:0 | 0.1889 | 0.0137 | 1.118142 | 0.762 (0.302) | 0.665 | 0.94 (0.41) | 0.821 |
| **lysoPC a C16:1** | lysoPhosphatidylcholine acyl C16:1 | 0.1972 | 0.5593 | 1.104777 | 4.154 (1.35) | 3.8 | 4.366 (2.276) | 4.055 |
| **SM C24:1** | Sphingomyeline C24:1 | 0.2208 | 0.1563 | 1.097326 | 62.854 (16.4) | 62 | 68.344 (22.2) | 66.1 |
| **C7-DC** | Pimelylcarnitine | 0.2324 | 0.6613 | 1.087345 | 0.054 (0.013) | 0.054 | 0.053 (0.024) | 0.047 |
| **PC aa C36:5** | Phosphatidylcholine diacyl C36:5 | 0.2453 | 0.1893 | 0.877061 | 46.72 (30.58) | 45.7 | 39.39 (20.83) | 35.288 |
| **lysoPC a C18:2** | lysoPhosphatidylcholine acyl C18:2 | 0.2556 | 0.0076 | 0.918123 | 42.42 (14.14) | 40.1 | 33.515 (18.6) | 30.140 |
| **PC aa C32:1** | Phosphatidylcholine diacyl C32:1 | 0.2584 | 0.5419 | 0.938549 | 23.17 (11.97) | 20.4 | 24.872 (15.9) | 20.969 |
| **SM C18:1** | Sphingomyeline C18:1 | 0.2586 | 0.1162 | 0.84321 | 13.672 (4.09) | 13.2 | 15.287 (6.16) | 14.776 |
| **PC ae C44:6** | Phosphatidylcholine acly-alkyl C44:6 | 0.2651 | 0.1162 | 1.092431 | 1.622 (0.538) | 1.55 | 1.444 (0.576) | 1.341 |
| **PC aa C34:4** | Phosphatidylcholine diacyl C34:4 | 0.2703 | 0.0406 | 1.096194 | 2.516 (0.882) | 2.37 | 2.106 (1.093) | 1.821 |
| **PC aa C36:6** | Phosphatidylcholine diacyl C36:6 | 0.2912 | 0.0627 | 0.926102 | 1.734 (0.847) | 1.65 | 2.157 (1.411) | 1.885 |
| **PC ae C38:5** | Phosphatidylcholine acly-alkyl C38:5 | 0.2912 | 0.0555 | 0.934282 | 23.76 (6.95) | 22.2 | 20.91 (7.627) | 19.7 |
| **lysoPC a C18:1** | lysoPhosphatidylcholine acyl C18:1 | 0.3058 | 0.7294 | 0.922655 | 32.31 (10.18) | 29.5 | 33.17 (14.93) | 29.9 |
| **His** | Histidine | 0.3293 | 0.4111 | 1.086674 | 110.83 (16.8) | 108 | 114.49 (27.7) | 113.880 |
| **C6 (C4:1-DC)** | Fumaryl-L-carnitine /Hexanoyl-Lcarnitine | 0.3324 | 0.3394 | 1.097853 | 0.095 (0.029) | 0.087 | 0.088 (0.036) | 0.076 |
| **C5:1** | Tiglylcarnitine | 0.3420 | 0.2260 | 0.934153 | 0.03 (0.008) | 0.029 | 0.028 (0.009) | 0.027 |
| **PC aa C36:1** | Phosphatidylcholine diacyl C36:1 | 0.3454 | 0.6253 | 0.932791 | 63.10 (17.76) | 62.4 | 65.05 (22.01) | 61.3 |
| **SM (OH) C16:1** | Hydroxysphingomyeline C16:1 | 0.3561 | 0.4618 | 0.934615 | 4.269 (1.16) | 4.09 | 4.478 (1.68) | 3.985 |
| **PC aa C36:4** | Phosphatidylcholine diacyl C36:4 | 0.3668 | 0.6198 | 0.92915 | 208.88 (50.1) | 207 | 215.34 (80.3) | 193.5 |
| **PC aa C36:0** | Phosphatidylcholine diacyl C36:0 | 0.3710 | 0.1134 | 0.938517 | 3.448 (1.462) | 3.01 | 4.085 (2.538) | 3.69 |
| **C14:2** | Tetradecadienyl carnitine | 0.3790 | 0.6168 | 0.931725 | 0.051 (0.02) | 0.047 | 0.053 (0.032) | 0.047 |
| **PC ae C32:2** | Phosphatidylcholine acly-alkyl C32:2 | 0.3807 | 0.2041 | 0.948319 | 0.844 (0.241) | 0.808 | 0.922 (0.372) | 0.880 |
| **PC aa C38:5** | Phosphatidylcholine diacyl C38:5 | 0.4100 | 0.3303 | 1.063594 | 80.03 (27.30) | 77.9 | 74.65 (26.55) | 68.9 |
| **SM C16:0** | Sphingomyeline C16:0 | 0.4296 | 0.4117 | 1.011639 | 124.62 (27.4) | 121 | 130.23 (41.1) | 126.24 |
| **PC aa C32:3** | Phosphatidylcholine diacyl C32:3 | 0.4411 | 0.3938 | 1.032985 | 0.729 (0.216) | 0.703 | 0.776 (0.328) | 0.730 |
| **C14:1-OH** | Hydroxytetradecenoyl carnitine | 0.4681 | 0.1186 | 1.045043 | 0.02 (0.005) | 0.019 | 0.022 (0.008) | 0.019 |
| **PC ae C36:2** | Phosphatidylcholine acly-alkyl C36:2 | 0.4781 | 0.5557 | 0.940581 | 18.79 (4.368) | 18.5 | 19.497 (7.58) | 18.940 |
| **PC ae C40:0** | Phosphatidylcholine acyl-alkyl C 40:0 | 0.4843 | 0.2323 | 1.048836 | 9.428 (3.023) | 8.45 | 8.699 (2.911) | 8.228 |
| **C12-DC** | Dodecanedioyl-L-carnitine | 0.4854 | 0.4029 | 1.087104 | 0.052 (0.003) | 0.052 | 0.053 (0.003) | 0.053 |
| **SM (OH) C22:1** | Hydroxysphingomyeline C22:1 | 0.5008 | 0.3454 | 1.059088 | 16.441 (4.34) | 15.6 | 15.43 (6.373) | 13.558 |
| **C14:1** | Tetradecenoyl carnitine | 0.5074 | 0.8774 | 0.968287 | 0.224 (0.06) | 0.212 | 0.222 (0.095) | 0.214 |
| **PC ae C34:2** | Phosphatidylcholine acly-alkyl C34:2 | 0.5108 | 0.1807 | 1.073364 | 13.643 (3.49) | 13.1 | 12.526 (4.83) | 11.768 |
| **PC aa C40:5** | Phosphatidylcholine diacyl C40:5 | 0.5467 | 0.4267 | 1.03749 | 14.32 (5.047) | 14 | 13.47 (5.515) | 12.8 |
| **Tyr** | Tyrosine | 0.5740 | 0.1891 | 1.050933 | 93.13 (18.84) | 91.4 | 87.41 (24.52) | 81.95 |
| **PC ae C44:5** | Phosphatidylcholine acly-alkyl C44:5 | 0.5939 | 0.3089 | 0.954248 | 2.301 (0.586) | 2.21 | 2.15 (0.903) | 2.115 |
| **SM (OH) C14:1** | Hydroxysphingomyeline C14:1 | 0.5965 | 0.2148 | 0.966331 | 8.318 (1.877) | 8.15 | 7.703 (3.069) | 7.285 |
| **PC aa C36:3** | Phosphatidylcholine diacyl C36:3 | 0.6356 | 0.9083 | 1.035091 | 164.42 (32.5) | 165 | 165.43 (55.7) | 156.5 |
| **SM (OH) C22:2** | Hydroxysphingomyeline C22:2 | 0.6364 | 0.5983 | 1.050833 | 13.15 (3.47) | 12.6 | 12.706 (4.94) | 11.85 |
| **C2** | Acetylcarnitine | 0.6453 | 0.8140 | 1.030918 | 8.901 (2.272) | 8.67 | 8.724 (5.132) | 7.765 |
| **C12** | Dodecanoylcarnitine | 0.6538 | 0.8587 | 1.030888 | 0.149 (0.048) | 0.137 | 0.147 (0.066) | 0.125 |
| **PC aa C38:0** | Phosphatidylcholine diacyl C38:0 | 0.6664 | 0.6606 | 0.964237 | 4.662 (1.76) | 4.45 | 4.495 (2.003) | 4.145 |
| **PC aa C42:6** | Phosphatidylcholine diacyl C42:6 | 0.6673 | 0.2563 | 0.970342 | 0.711 (0.215) | 0.713 | 0.773 (0.327) | 0.778 |
| **PC ae C40:6** | Phosphatidylcholine acly-alkyl C40:6 | 0.6681 | 0.3408 | 0.978785 | 7.117 (2.467) | 6.84 | 6.613 (2.763) | 5.942 |
| **SM (OH) C24:1** | Hydroxysphingomyeline C24:1 | 0.6853 | 0.6837 | 0.972736 | 1.632 (0.442) | 1.65 | 1.587 (0.652) | 1.467 |
| **C6:1** | Hexenoylcarnitine | 0.7077 | 0.7108 | 1.031623 | 0.022 (0.005) | 0.022 | 0.022 (0.005) | 0.021 |
| **C14:2-OH** | Hydroxytetradecadienylcarnitine | 0.7157 | 0.9219 | 0.981402 | 0.011 (0.003) | 0.01 | 0.011 (0.003) | 0.01 |
| **PC aa C30:0** | Phosphatidylcholine diacyl C30:0 | 0.7157 | 0.1591 | 1.026838 | 4.804 (1.741) | 4.55 | 4.213 (2.439) | 3.608 |
| **PC aa C28:1** | Phosphatidylcholine diacyl C28:1 | 0.7256 | 0.3780 | 0.984288 | 3.691 (0.803) | 3.72 | 3.5 (1.359) | 3.345 |
| **C4:1** | Butenylcarnitine | 0.7297 | 0.6803 | 0.987399 | 0.015 (0.002) | 0.015 | 0.015 (0.004) | 0.013 |
| **PC aa C38:3** | Phosphatidylcholine diacyl C38:3 | 0.7516 | 0.9773 | 0.98015 | 63.59 (17.69) | 64.2 | 63.70 (22.18) | 61.253 |
| **C8:1** | Octenoyl-L-carnitine | 0.8296 | 0.4798 | 0.98632 | 0.232 (0.118) | 0.192 | 0.252 (0.169) | 0.199 |
| **PC ae C32:1** | Phosphatidylcholine acly-alkyl C32:1 | 0.8431 | 0.8045 | 1.009891 | 3.176 (0.794) | 3.04 | 3.126 (1.227) | 2.93 |
| **PC ae C30:0** | Phosphatidylcholine acly-alkyl C30:0 | 0.8488 | 0.9680 | 1.008371 | 0.45 (0.124) | 0.433 | 0.448 (0.242) | 0.407 |
| **PC aa C38:4** | Phosphatidylcholine diacyl C38:4 | 0.8605 | 0.9100 | 0.989363 | 135.77 (37.6) | 127 | 134.78 (49.9) | 125.846 |
| **SM C24:0** | Sphingomyeline C24:0 | 0.8710 | 0.8718 | 1.006178 | 29.229 (7.2) | 30.2 | 29.52 (10.76) | 27.85 |
| **C9** | Nonaylcarnitine | 0.8864 | 0.3594 | 0.992709 | 0.038 (0.012) | 0.036 | 0.035 (0.016) | 0.031 |
| **PC aa C36:2** | Phosphatidylcholine diacyl C36:2 | 0.8868 | 0.5136 | 0.994422 | 264.71 (44.8) | 260 | 256.31 (83.3) | 251.498 |
| **H1** | Hexose | 0.9486 | 0.8753 | 0.996727 | 6132 (937.4) | 6044 | 6183.33 (2269) | 5877.5 |
| **SM C16:1** | Sphingomyeline C16:1 | 0.9687 | 0.9959 | 1.001786 | 19.983 (4.71) | 19.5 | 19.989 (6.34) | 19.55 |
| **SM C26:1** | Sphingomyeline C26:1 | 0.9787 | 0.5650 | 1.000285 | 0.468 (0.172) | 0.441 | 0.446 (0.196) | 0.419 |

**Supplementary Table 2**. List of 40 metabolites with Kruskal-Wallis inter-group *p*-value < 0.30 in targeted MS/MS analysis of stages 1, 2 and 3 locoregional colorectal cancer (PC: Phosphatidylcholine, Lyso-PC: lysophosphatidylcholine, ae: acyl-alkyl, aa: diacyl, SM: sphingomyelin)

| **Short name** | **Metabolite** | ***p*-value** |
| --- | --- | --- |
| **PC aa C36:6** | Phosphatidylcholine diacyl C36:6 | 0.031 |
| **PC ae C36:1** | Phosphatidylcholine acyl-alkyl C36:1 | 0.045 |
| **Val** | Valine | 0.061 |
| **PC aa C34:4** | Phosphatidylcholine diacyl C34:4 | 0.075 |
| **PC aa C34:3** | Phosphatidylcholine diacyl C34:3 | 0.089 |
| **C7-DC** | Pimelylcarnitine | 0.097 |
| **xLeu** | Leucine/Isoleucine | 0.119 |
| **PC aa C38:3** | Phosphatidylcholine diacyl C38:3 | 0.129 |
| **Met** | Methionine | 0.148 |
| **Trp** | Tryptophan | 0.148 |
| **C18:2** | Octadecadienylcarnitine | 0.151 |
| **PC ae C44:5** | Phosphatidylcholine acly-alkyl C44:5 | 0.164 |
| **Tyr** | Tyrosine | 0.164 |
| **SM (OH) C22:2** | Hydroxysphingomyeline C22:2 | 0.165 |
| **PC aa C28:1** | Phosphatidylcholine diacyl C28:1 | 0.165 |
| **PC ae C44:6** | Phosphatidylcholine acyl-alkyl C44:6 | 0.179 |
| **PC ae C40:1** | Phosphatidylcholine acyl-alkyl C40:1 | 0.195 |
| **PC ae C38:0** | Phosphatidylcholine acyl-alkyl C38:0 | 0.206 |
| **PC ae C38:3** | Phosphatidylcholine acyl-alkyl C38:3 | 0.206 |
| **SM (OH) C16:1** | Hydroxysphingomyeline C16:1 | 0.221 |
| **PC ae C36:5** | Phosphatidylcholine acyl-alkyl C36:5 | 0.222 |
| **Gln** | Glutamine | 0.229 |
| **PC aa C42:1** | Phosphatidylcholine diacyl C42:1 | 0.229 |
| **PC ae C36:2** | Phosphatidylcholine acyl-alkyl C36:2 | 0.235 |
| **PC ae C30:0** | Phosphatidylcholine acyl-alkyl C30:0 | 0.235 |
| **SM (OH) C14:1** | Hydroxysphingomyeline C14:1 | 0.242 |
| **PC ae C42:2** | Phosphatidylcholine acyl-alkyl C42:2 | 0.242 |
| **SM (OH) C22:1** | Hydroxysphingomyeline C22:1 | 0.247 |
| **PC ae C40:2** | Phosphatidylcholine acyl-alkyl C40:2 | 0.250 |
| **PC ae C38:5** | Phosphatidylcholine acyl-alkyl C38:5 | 0.2645 |
| **PC aa C42:6** | Phosphatidylcholine diacyl C42:6 | 0.264 |
| **PC aa C42:2** | Phosphatidylcholine diacyl C42:2 | 0.275 |
| **PC ae C44:3** | Phosphatidylcholine acyl-alkyl C44:3 | 0.278 |
| **lysoPC a C17:0** | lysoPhosphatidylcholine acyl C17:0 | 0.278 |
| **PC ae C42:5** | Phosphatidylcholine acyl-alkyl C42:5 | 0.278 |
| **PC aa C36:5** | Phosphatidylcholine diacyl C36:5 | 0.281 |
| **C18** | Octadecanoylcarnitine | 0.281 |
| **PC ae C42:1** | Phosphatidylcholine acyl-alkyl C42:1 | 0.281 |
| **PC aa C36:1** | Phosphatidylcholine diacyl C36:1 | 0.287 |
| **PC ae C42:4** | Phosphatidylcholine acyl-alkyl C42:4 | 0.287 |

**Supplementary Table 3**. List of 75 metabolites with Welch’s inter-group *p*-value < 0.30 in the targeted analysis of colorectal adenoma vs. matched controls; red indicates relative increase and green indicates a relative decrease. (PC: Phosphatidylcholine, Lyso-PC: lysophosphatidylcholine, ae: acyl-alkyl, aa: diacyl, SM: sphingomyelin)

| **Metabolite** | **Metabolite name** | **Control** | **Adenoma** | **Fold change** | ***p*-value** |
| --- | --- | --- | --- | --- | --- |
| **Pro** | Proline | 149.676 (30.372) | 183.41 (50.02) | 1.23 | 0.0027 |
| **C14:2** | Tetradecadienylcarnitine | 0.05 (0.022) | 0.037 (0.016) | 0.73 | 0.0081 |
| **Trp** | Tryptophan | 80.098 (13.029) | 88.339 (12.136) | 1.1 | 0.0139 |
| **C12:1** | Dodecenoylcarnitine | 0.191 (0.074) | 0.15 (0.052) | 0.79 | 0.0173 |
| **C10:1** | Decenoylcarnitine | 0.225 (0.076) | 0.185 (0.064) | 0.82 | 0.0306 |
| **C16:2** | Hexadecadienylcarnitine | 0.016 (0.005) | 0.014 (0.004) | 0.84 | 0.0319 |
| **C14:1-OH** | Hydroxytetradecenoylcarnitine | 0.019 (0.006) | 0.016 (0.004) | 0.84 | 0.0329 |
| **PC ae C40:2** | Phosphatidylcholine acly-alkyl C40:2 | 2.769 (0.765) | 3.226 (0.869) | 1.16 | 0.0349 |
| **lysoPC a C17:0** | lysoPhosphatidylcholine acyl C17:0 | 1.5 (0.367) | 1.789 (0.652) | 1.19 | 0.0399 |
| **PC aa C34:4** | Phosphatidylcholine diacyl C34:4 | 3.091 (1.439) | 3.908 (1.678) | 1.26 | 0.0477 |
| **C3** | Propionylcarnitine | 0.332 (0.075) | 0.381 (0.113) | 1.15 | 0.051 |
| **C12** | Dodecanoylcarnitine | 0.154 (0.061) | 0.126 (0.049) | 0.82 | 0.0515 |
| **PC ae C34:0** | Phosphatidylcholine acly-alkyl C34:0 | 2.165 (0.824) | 2.571 (0.771) | 1.19 | 0.0531 |
| **C7-DC** | Pimelylcarnitine | 0.046 (0.017) | 0.038 (0.015) | 0.82 | 0.0548 |
| **PC ae C38:3** | Phosphatidylcholine acly-alkyl C38:3 | 5.265 (1.087) | 5.847 (1.248) | 1.11 | 0.0589 |
| **C6 (C4:1-DC)** | Hexanoyl-L-carnitine | 0.088 (0.029) | 0.075 (0.023) | 0.85 | 0.0658 |
| **C10** | Decanoylcarnitine | 0.404 (0.175) | 0.327 (0.149) | 0.81 | 0.0687 |
| **PC ae C38:4** | Phosphatidylcholine acly-alkyl C38:4 | 18.658 (4.443) | 20.674 (4.051) | 1.11 | 0.0713 |
| **C14:1** | Tetradecenoylcarnitine | 0.151 (0.055) | 0.129 (0.037) | 0.85 | 0.0742 |
| **PC aa C36:6** | Phosphatidylcholine diacyl C36:6 | 1.495 (0.927) | 1.918 (0.876) | 1.28 | 0.0743 |
| **PC aa C32:2** | Phosphatidylcholine diacyl C32:2 | 4.496 (2.451) | 5.644 (2.677) | 1.26 | 0.0885 |
| **PC ae C36:5** | Phosphatidylcholine acly-alkyl C36:5 | 17.427 (5.658) | 20.142 (6.657) | 1.16 | 0.094 |
| **C8** | Octanoylcarnitine | 0.263 (0.103) | 0.222 (0.088) | 0.84 | 0.0945 |
| **PC ae C36:1** | Phosphatidylcholine acly-alkyl C36:1 | 11.395 (3.027) | 12.639 (2.636) | 1.11 | 0.0948 |
| **PC aa C30:0** | Phosphatidylcholine diacyl C30:0 | 3.765 (2.025) | 4.673 (2.168) | 1.24 | 0.0989 |
| **xLeu** | Leucine/Isoleucine | 259.639 (43.721) | 280.80 (53.85) | 1.08 | 0.1002 |
| **C18:1** | Octadecenoylcarnitine | 0.23 (0.062) | 0.205 (0.053) | 0.89 | 0.1018 |
| **lysoPC a C16:0** | lysoPhosphatidylcholine acyl C16:0 | 81.11 (17.777) | 89.3 (20.322) | 1.1 | 0.1019 |
| **PC aa C38:4** | Phosphatidylcholine diacyl C38:4 | 172.968 (44.429) | 191.28 (42.34) | 1.11 | 0.1075 |
| **C16:1** | Hexadecenoyl-L-carnitine | 0.058 (0.018) | 0.052 (0.011) | 0.89 | 0.1111 |
| **PC aa C36:4** | Phosphatidylcholine diacyl C36:4 | 284.246 (76.065) | 315.11 (72.77) | 1.11 | 0.1136 |
| **PC aa C38:5** | Phosphatidylcholine diacyl C38:5 | 101.733 (36.582) | 115.99 (32.19) | 1.14 | 0.1144 |
| **PC ae C40:6** | Phosphatidylcholine acly-alkyl C40:6 | 7.765 (2.157) | 8.854 (3.035) | 1.14 | 0.1151 |
| **Orn** | Ornithine | 77.583 (17.132) | 84.175 (15.318) | 1.08 | 0.1215 |
| **PC ae C38:0** | Phosphatidylcholine acly-alkyl C38:0 | 4.056 (1.781) | 4.756 (1.744) | 1.17 | 0.1298 |
| **PC ae C34:1** | Phosphatidylcholine acly-alkyl C34:1 | 14.156 (3.744) | 15.604 (3.598) | 1.1 | 0.1318 |
| **C2** | Acetylcarnitine | 10.492 (3.74) | 8.929 (4.204) | 0.85 | 0.1333 |
| **PC ae C38:6** | Phosphatidylcholine acly-alkyl C38:6 | 12.804 (4.488) | 14.661 (5.138) | 1.14 | 0.1415 |
| **C18:2** | Octadecadienylcarnitine | 0.095 (0.03) | 0.085 (0.022) | 0.9 | 0.1473 |
| **PC ae C30:0** | Phosphatidylcholine acly-alkyl C30:0 | 0.347 (0.147) | 0.398 (0.119) | 1.15 | 0.1485 |
| **C0** | Carnitine | 42.225 (8.022) | 45.549 (9.856) | 1.08 | 0.1573 |
| **PC aa C40:6** | Phosphatidylcholine diacyl C40:6 | 39.256 (12.892) | 44.454 (15.557) | 1.13 | 0.1641 |
| **PC aa C38:6** | Phosphatidylcholine diacyl C38:6 | 132.996 (47.314) | 150.48 (49.28) | 1.13 | 0.1662 |
| **PC aa C36:5** | Phosphatidylcholine diacyl C36:5 | 52.181 (38.279) | 64.601 (30.791) | 1.24 | 0.1715 |
| **PC ae C36:2** | Phosphatidylcholine acly-alkyl C36:2 | 18.94 (4.401) | 20.572 (4.737) | 1.09 | 0.1722 |
| **PC aa C28:1** | Phosphatidylcholine diacyl C28:1 | 3.727 (1.109) | 4.11 (1.04) | 1.1 | 0.1728 |
| **PC aa C34:1** | Phosphatidylcholine diacyl C34:1 | 333.79 (95.553) | 369.089 (103.529) | 1.11 | 0.1751 |
| **PC aa C32:3** | Phosphatidylcholine diacyl C32:3 | 0.739 (0.281) | 0.834 (0.262) | 1.13 | 0.1821 |
| **Met** | Methionine | 32.569 (6.315) | 34.768 (6.549) | 1.07 | 0.1905 |
| **PC ae C40:5** | Phosphatidylcholine acly-alkyl C40:5 | 5.484 (1.329) | 5.957 (1.446) | 1.09 | 0.1919 |
| **lysoPC a C28:1** | lysoPhosphatidylcholine acyl C28:1 | 0.433 (0.121) | 0.469 (0.085) | 1.08 | 0.1925 |
| **PC ae C36:4** | Phosphatidylcholine acly-alkyl C36:4 | 24.724 (8.354) | 27.248 (6.379) | 1.1 | 0.1938 |
| **SM C16:1** | Sphingomyeline C16:1 | 19.109 (3.967) | 20.468 (4.077) | 1.07 | 0.1956 |
| **Thr** | Threonine | 107.824 (23.563) | 115.90 (24.44) | 1.07 | 0.1975 |
| **PC aa C32:1** | Phosphatidylcholine diacyl C32:1 | 23.549 (17.126) | 31.96 (30.944) | 1.36 | 0.1991 |
| **C10:2** | Decadienylcarnitine | 0.052 (0.015) | 0.047 (0.011) | 0.91 | 0.1994 |
| **Arg** | Arginine | 117.083 (20.274) | 124.43 (23.46) | 1.06 | 0.1996 |
| **PC aa C32:0** | Phosphatidylcholine diacyl C32:0 | 20.422 (6.313) | 22.411 (5.911) | 1.1 | 0.2126 |
| **His** | Histidine | 107.172 (14.598) | 112.43 (17.586) | 1.05 | 0.2128 |
| **SM (OH) C14:1** | Hydroxysphingomyeline C14:1 | 7.027 (2.036) | 7.632 (1.776) | 1.09 | 0.2248 |
| **Tyr** | Tyrosine | 82.831 (18.043) | 89.28 (22.693) | 1.08 | 0.2281 |
| **PC aa C34:2** | Phosphatidylcholine diacyl C34:2 | 544.448 (98.524) | 574.80 (97.414) | 1.06 | 0.2348 |
| **lysoPC a C18:0** | lysoPhosphatidylcholine acyl C18:0 | 26.962 (5.771) | 29.004 (7.478) | 1.08 | 0.2414 |
| **PC ae C36:0** | Phosphatidylcholine acly-alkyl C36:0 | 1.253 (0.446) | 1.42 (0.636) | 1.13 | 0.2449 |
| **lysoPC a C16:1** | lysoPhosphatidylcholine acyl C16:1 | 2.368 (1.022) | 2.794 (1.735) | 1.18 | 0.2522 |
| **PC ae C40:1** | Phosphatidylcholine acly-alkyl C40:1 | 2.338 (0.704) | 2.549 (0.719) | 1.09 | 0.2545 |
| **PC aa C36:0** | Phosphatidylcholine diacyl C36:0 | 3.473 (1.484) | 3.983 (2.001) | 1.15 | 0.2674 |
| **PC ae C32:2** | Phosphatidylcholine acly-alkyl C32:2 | 0.904 (0.278) | 0.989 (0.309) | 1.09 | 0.2688 |
| **PC aa C42:6** | Phosphatidylcholine diacyl C42:6 | 0.541 (0.215) | 0.601 (0.205) | 1.11 | 0.2695 |
| **PC ae C42:2** | Phosphatidylcholine acly-alkyl C42:2 | 0.772 (0.224) | 0.838 (0.237) | 1.09 | 0.2721 |
| **PC aa C38:3** | Phosphatidylcholine diacyl C38:3 | 69.871 (16.146) | 74.793 (18.819) | 1.07 | 0.2814 |
| **C4** | Butyrylcarnitine | 0.245 (0.078) | 0.224 (0.071) | 0.91 | 0.2832 |
| **PC ae C40:0** | Phosphatidylcholine acyl-alkyl C40:0 | 11.179 (3.357) | 12.215 (4.028) | 1.09 | 0.2834 |
| **SM (OH) C24:1** | Hydroxysphingomyeline C24:1 | 1.913 (0.533) | 2.051 (0.478) | 1.07 | 0.2974 |
| **PC aa C36:1** | Phosphatidylcholine diacyl C36:1 | 79.908 (23.452) | 86.598 (25.931) | 1.08 | 0.2988 |
