# Supplementary figures and images for: A quantitative multimodal metabolomic assay for colorectal cancer
Source: BMC Cancer. 2018 Jan 4;18:26. doi: 10.1186/s12885-017-3923-z (PMC5755335; doi:10.1186/s12885-017-3923-z)

## Slide 1
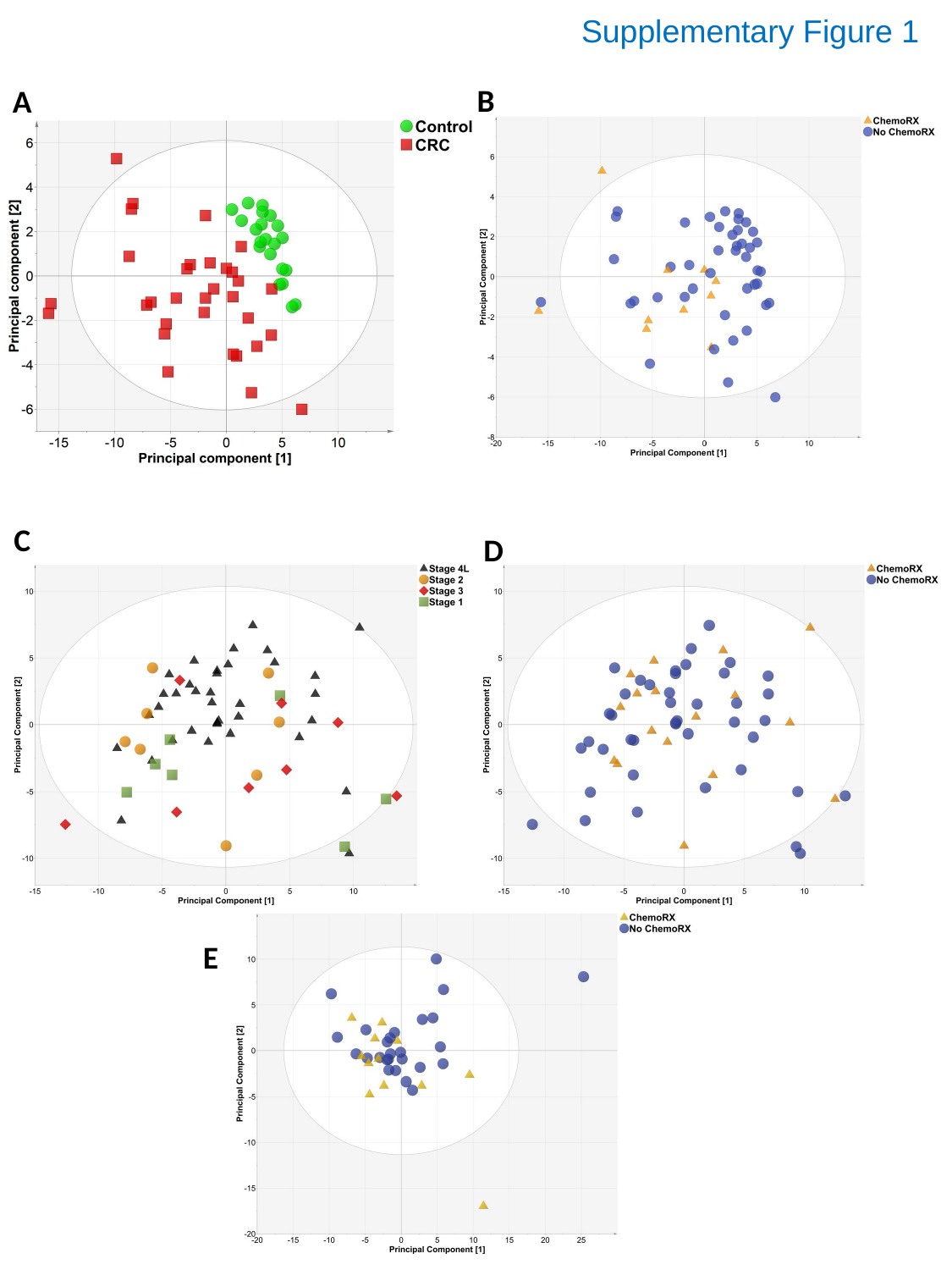

Supplementary Figure 1
B
A
C
D
E

Supplement: Supplementary file 2 — Metabolomic profile of CRC, Stages I to IVa by OPLS-DA supervised analysis. A. Scores scatter plot of discriminant analysis. Model characteristics are indicated. The first component clearly distinguishes between CRC and control groups, while the second component identifies locoregional CRC from liver-metastatic CRC (stage IVa). (PPTX 8816 kb) [file 12885_2017_3923_MOESM2_ESM.pptx]

## Slide 1
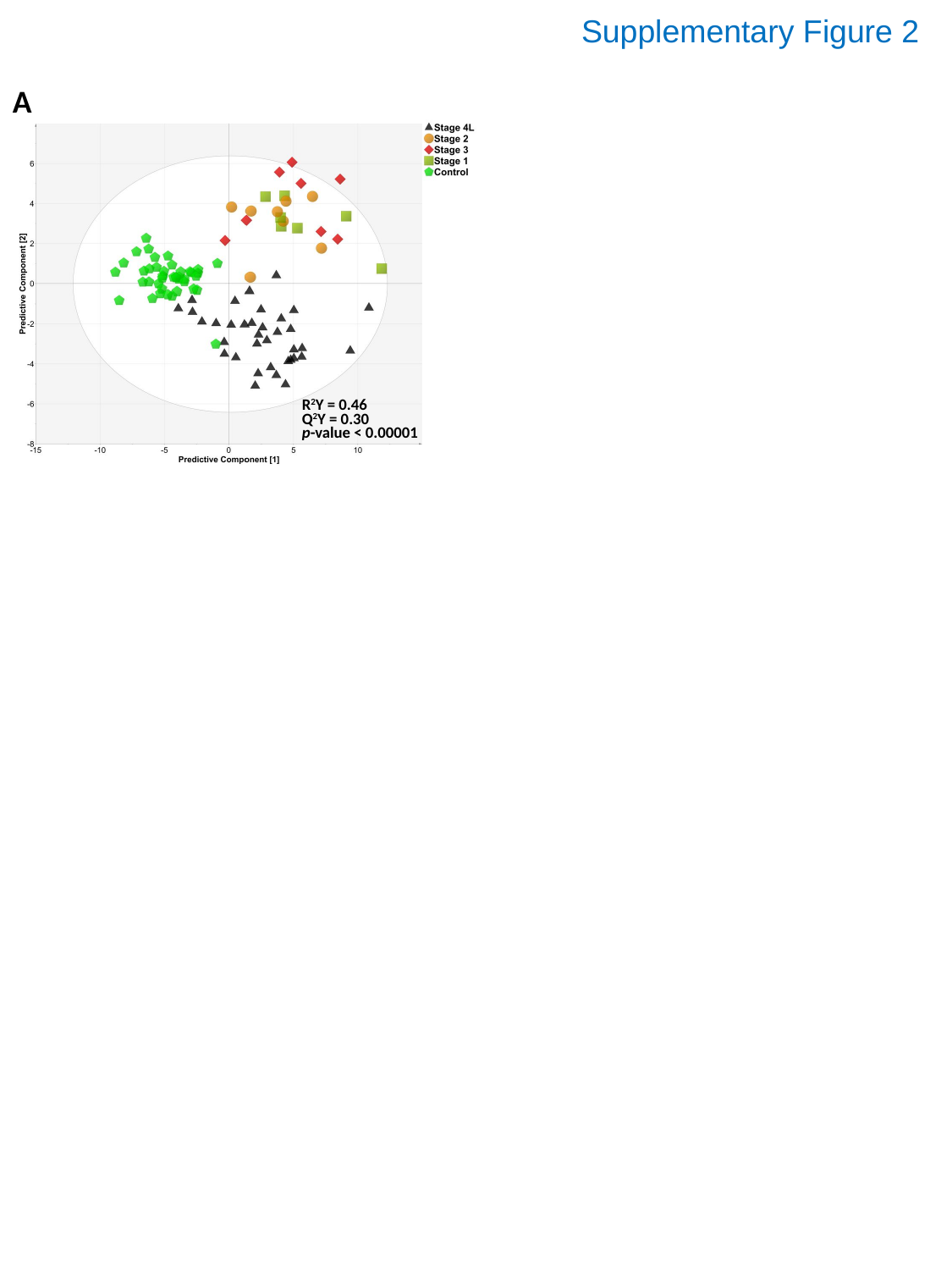

Supplementary Figure 2
A
R2Y = 0.46
Q2Y = 0.30
p-value < 0.00001

Supplement: Supplementary file 3 — S2 and S3. Lists of metabolites incorporated into each metabolomic signature for colorectal cancer and colorectal adenoma (PPTX 2288 kb) [file 12885_2017_3923_MOESM3_ESM.pptx]
